# Converting unstructured cardiac catheterization and echocardiography reports into structured data using transformer-based language models

**DOI:** 10.1093/jamiaopen/ooag036

**Published:** 2026-03-26

**Authors:** Fagen Xie, Ming-Sum Lee, Wansu Chen, Derek Q Phan

**Affiliations:** Department of Research and Evaluation, Kaiser Permanente Southern California Medical Group, Pasadena, CA 91101, United States; Department of Cardiology, Kaiser Permanente Los Angeles Medical Center, Los Angeles, CA 90027, United States; Department of Research and Evaluation, Kaiser Permanente Southern California Medical Group, Pasadena, CA 91101, United States; Regional Cardiac Catheterization Lab, Kaiser Permanente Los Angeles Medical Center, Los Angeles, CA 90027, United States

**Keywords:** transformer-based language model, BioclinicalBERT, BART-Large-CNN, unstructured data, echocardiography, cardiac catheterization

## Abstract

**Objectives:**

Echocardiography and cardiac catheterization reports capture important clinical assessment information of cardiac function and disease severity. This study explores using open-source transformer-based language models (LMs) that are run locally within an institutional environment as a privacy-preserving alternative to external API-based large LM to systematically extract clinical data from unstructured echocardiography and cardiac catheterization reports, aiming to improve data accessibility for research and patient care.

**Materials and Methods:**

Two transformer-based LMs, BioclinicalBERT and BART-Large-CNN, were fine-tuned in a secure local environment using a question-answering approach. The dataset included 3286 echocardiography and 1884 cardiac catheterization reports from Kaiser Permanente Southern California’s electronic health records, annotated for 25 and 47 predefined categories, respectively. Three hundred reports from each type were randomly selected and used for validation, with the remainder for training. Model performance was assessed using accuracy, precision, recall, and F1-score at 2 probability thresholds. The effect of training set size on model performance was also evaluated.

**Results:**

Both models achieved consistent and high accuracy, precision, and recall (all >90%) across the 5 seed runs for both report types. For echocardiography, BioclinicalBERT reached mean accuracy of 95.7%, precision of 97.6%, recall of 97.4%, and F1-score of 0.98 at the probability threshold of 0.1; BART-Large-CNN had similar results. For cardiac catheterization, BART-Large-CNN slightly outperformed BioclinicalBERT with mean accuracy 94.9% vs 94.3%; precision 96.7% vs 96.3%; recall 96.1% vs 95.7%, and F1-score 0.96 vs 0.96 at the probability threshold of 0.1. Most individual categories showed strong performance, though a few (eg, prosthetic mitral valve, right atrial pressure) had lower scores. Performance improved with more training data, but plateauing around 1000 reports.

**Discussion and conclusion:**

Fine-tuned transformer-based LMs can effectively extract structured data from unstructured cardiac reports, supporting automated information extraction to enhance research and clinical applications.

## Background and significance

Echocardiography and cardiac catheterization reports contain detailed and clinically important information regarding cardiac structure, function, and hemodynamics.^1–4^ However, these data are frequently documented in semistructured or complex free-text formats within electronic health record (EHR) systems, limiting their accessibility, standardization, and secondary use for clinical care and research. This lack of structure poses a significant barrier to large-scale analysis and necessitates the use of advanced methods to transform narrative content into structured, analyzable data.^5^^,^^6^

Over the past several years, many studies have explored the extraction of clinically relevant information from echocardiography reports using rule-based or machine learning-based natural language processing approaches.^7–10^ More recently, advanced transformer-based large language models (LLMs) have emerged as powerful tools for converting unstructured text into structured data and have been extensively evaluated across diverse domains.^11–18^ However, the majority of studies have relied on pretrained large-scale LLMs hosted on third-party external servers and accessed via application programming interfaces, raising substantial security, privacy, and regulatory compliance issues, particularly when applied to EHRs. An alternative and increasingly viable approach is to implement and fine-tune LLMs within a standalone local computing environment.^19^ However, to the best of our knowledge, no prior studies have systematically extracted information from both echocardiography and cardiac catheterization reports using LLMs approach.

### Objective

In this study, we evaluated the feasibility of converting clinical assessment information from unstructured echocardiography and cardiac catheterization reports into structured data. To achieve this objective, we implemented and fine-tuned 2 prominent open-source transformer-based language models (LMs) within a secure computing environment at Kaiser Permanente Southern California (KPSC): BioClinicalBERT,^20^ which is extensively pretrained on clinical notes, and BART-Large-CNN,^21^ which is heavily pretrained on CNN Daily Mail articles. By systematically comparing the performance of these models, our findings can inform us of the optimal selection of LMs for practical downstream applications.

## Materials and methods

**Figure 1.**
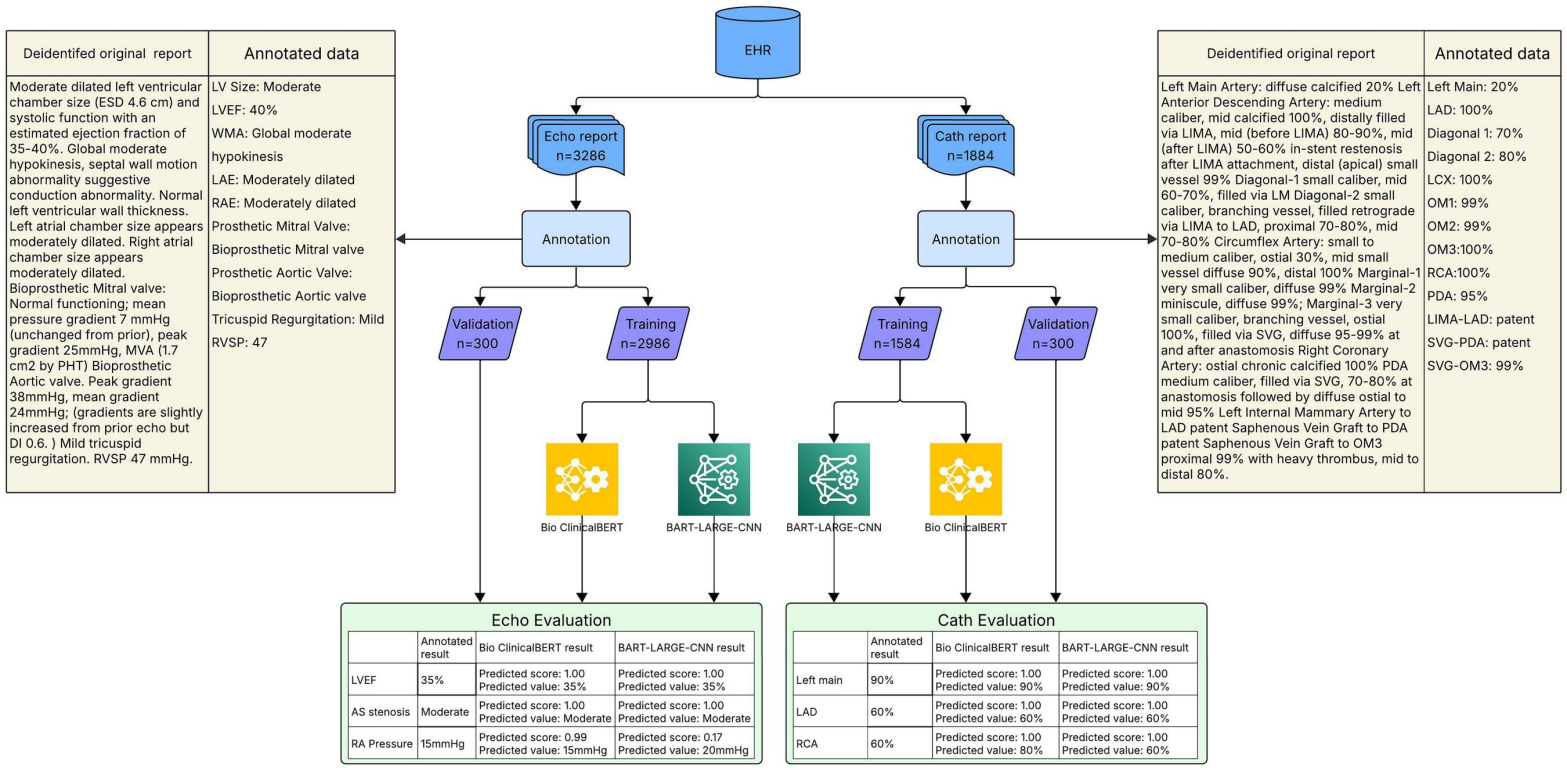
Schematic diagram describing the overall process for extracting information from echocardiography and cardiac catheterization reports using the BioclinicalBERT and BART-Large-CNN models.

A question-answering framework was employed to fine-tune the transformer-based LMs for the extraction of documented measurements from echocardiography and cardiac catheterization reports. The overall processing flow is shown in Figure 1. Each step is described in detail below.

### Data sources

Patients who underwent echocardiography or cardiac catheterization within KPSC from June 1, 2013, to August 31, 2024, were identified. The corresponding detailed reports were extracted from the KPSC EHR system’s back-end database with free-text format. A total of 3286 echocardiography reports and 1884 cardiac catheterization reports were randomly selected from these extracted reports to form the study datasets. These reports are sufficient for the development of LLM applications.^22^ Each selected cardiac catheterization report belonged to a different patient, whereas multiple echocardiography reports could come from the same patient, but typically occurred months to years apart. These reports were preprocessed to remove the nonclinical information (eg, procedure title and location, patient information and physician signature) for manual annotations.

### Data annotations

Cardiologists (D.Q.P. and M.-S.L.) identified 25 clinically relevant categories for echocardiography reports (Table S1) and 47 categories for catheterization reports (Table S2). These categories captured the majority of disease-related and quantitative measurements routinely documented in echocardiography and cardiac catheterization reports. One study cardiologist (D.Q.P.) manually reviewed each report and constructed structured data in JSON format, recording values for each predefined category. A custom graphical interface was used to assist the annotations in Python using the Tkinter library. The tool loaded the extracted cardiac catheterization and echocardiogram reports and presented each report in a graphical viewer. This tool allowed manual highlighting and selecting of text spans to assign it a label (coronary segment: left main, left anterior descending artery (LAD), etc., or echo field: left ventricular ejection fraction (LVEF), aortic stenosis, etc.). Each label annotation was stored with character level start/end offsets, the selected label, and highlighted text verbatim from the report, then were exported to a JSON, enabling consistent labels. The output annotated reports were also double examined to guarantee consistency with designed texts for each label in the graphical tool and each label with a unique assigned value. If the category was not mentioned in the report, the corresponding value was recorded as “Null.” Each annotated record included: (1) the full report text; (2) the target label category (eg, left ventricle ejection fraction, aortic stenosis, left anterior descending artery, etc.); (3) the extracted answer text span in the report, and (4) the start and end positions of the answer text within the report.

### Training dataset and validation dataset

For echocardiography reports, 300 annotated reports were randomly selected at report level and held out for performance evaluation. The remaining 2986 annotated echocardiography reports were used for transformer-based LM’s fine-tuning. For cardiac catheterization reports, 1584 reports were first sampled for training dataset requiring some labeled category described in the reports to guarantee the representative samples for effective training and the remaining reports were used for validation.

The annotated training datasets were further split into train/internal validation sets (0.9 vs 0.1) for each report type and models. Subsets randomly selected from training datasets were also constructed to investigate the relationship between training set size and model performance.

### Model development

We used 2 representative transformer-based open-source deep learning models: BioclinicalBERT, pretrained on clinical notes,^20^ and BART-Large-CNN, pretrained on the English language and fine-tuned on CNN Daily Mail,^21^ as our base models. Both models were downloaded from the Hugging Face Artificial Intelligence (AI) community repository^23^^,^^24^ and installed within a secure local computing environment, and fine-tuned on the annotated training datasets using a question-answering framework via the transformer library within pytorch environment, with one question per target label (eg, “What is the most severe disease in the left main artery?”, “What is the left ventricle ejection refraction?”, etc.). Hyperparameters included a learning rate of 2 × 10^−5^, maximum sequence length of 512, warmup fraction of 6%, batch size of 4 and rand seeds of 42, 736, 299, 971, 243. Training was conducted across multiple epochs, with both models achieving stable and optimal performance at the eighth epoch.

### Model evaluation

The fine-tuned BioclinicalBERT and BART-Large-CNN models run against the validation datasets to generate the values and corresponding probability score for each category or label. For each report type and model, performance of the model extracted values was evaluated against the manual annotated results of the validation datasets for overall and each label category using accuracy, positive predictive value (PPV; precision), sensitivity (recall), and F1-score at 2 selected probability thresholds (0.1 and 0.5). Accuracy was defined as the number of cases in which the model and annotator extracted exactly matching values divided by the total number of retrieved cases (annotator or model). Recall was defined as the number of cases with exactly matching values extracted by both the annotator and the model, divided by the total number of cases retrieved by the annotator. Precision was defined as the number of cases exactly matching values extracted by both the annotator and the model, divided by the total number of cases retrieved by the model. F1-score was calculated as: 2×PPV×sensitivity/(PPV+sensitivity). The mean and SD of the performance among the models trained on 5 different rand seeds was also calculated.

### Discrepancy analysis

Discordant results between the model outputs and annotator-derived labels in the validation datasets were systematically analyzed and summarized in detail.

### Model performance as a function of the number of training reports

For each report type, the dependence of the model’s performance on the number of training reports at the probability threshold of 0.1 was investigated and reported in detail.

### Computational environment

Model development and analyses were conducted using Python 3.10 (Python Software Foundation). Model training was performed on a Lambda workstation equipped with 1 TB RAM, an AMD Threadripper Pro 3975WX 32-core 3.50 GHz CPUs, and 4 RTX A6000 GPUs (49 GB RAM each).

### Ethics approval

The KPSC Institutional Review Board reviewed and approved the study protocol with a waiver of the requirement for informed consent (approval numbers: 13954 and 14415). The study was conducted in compliance with the Health Insurance Portability and Accountability Act. All analyses were performed by authorized personnel with appropriate access permissions.

## Results

### Distribution of annotated labeled categories

The frequency of each labeled category with annotated values regarding the severity in both training and validation datasets are summarized in Table S3 for echocardiography report and Table S4 for cardiac catheterization reports. The most 3 frequently labeled categories were LVEF (2802 in training, 281 in validation), LV size (1972 in training, 198 in validation), and pericardial effusion (1849 in training, 189 in validation) among 2986 training and 300 validation echocardiography reports, and left main (1519 in training, 285 in validation), LAD (1518 in training, 289 in validation), right coronary artery (RCA) (1503 in training, 277 in validation) among 1584 training and 300 validation cardiac catheterization reports. Most of combined labeled categories (eg, left internal mammary artery-obtuse marginal (LIMA-OM), saphenous vein graft-left circumflex coronary artery (SVG-LCX)) in cardiac catheterization reports had a small or zero case in both training and validation datasets.

### Model performance metrics

For echocardiography reports, the performance of the fine-tuned BioclinicalBERT and BART-Large-CNN at rand seed of 42 on the validation dataset at probability thresholds of 0.1 and 0.5 is presented in Tables 1 and 2, respectively. Both models achieved comparable results in terms of overall performance as well as for each label category. Specifically, BioclinicalBERT yielded an overall accuracy of 95.78%, precision of 97.74%, recall of 97.39%, and F1-score of 0.98 at a probability threshold of 0.1; and accuracy of 95.48%, precision of 97.86%, recall of 97.04%, and F1-score of 0.97 at a probability threshold of 0.5. BART-Large-CNN yielded an overall accuracy of 95.57%, precision of 97.86%, recall of 97.21%, and F1-score of 0.98 at the probability threshold of 0.1, and accuracy of 95.30%, precision of 97.94%, recall of 96.69%, and F1-score of 0.97 at the probability threshold of 0.5, respectively. For each label category, both models demonstrated consistently high performance, with accuracy, precision, and recall exceeding 90% and F1-scores greater than 0.90 for most categories. Exceptions included prosthetic mitral valve, pulmonic stenosis and right atrial pressure, for which performance was comparatively lower. Similar performance of the 2 models at the other rand seeds are presented in Table S5. The mean and SD of the performance across the 5 runs are summarized in Table S6. The BioclinicalBERT model reached mean accuracy of 95.7%, precision of 97.6%, recall of 97.4%, and F1-score of 0.98 at the probability threshold of 0.1; and mean accuracy of 95.4%, precision of 97.7%, recall of 97.1%, and F1-score of 0.97 at the probability threshold of 0.5. The BART-Large-CNN model had similar mean performance.

**Table 1.** The performance of the fine-tuned BioclinicalBERT model against the manual annotated results in the echocardiography validation report dataset (*n* = 300) at 0.1 and 0.5 cutoff thresholds of probability.

Extracted outcome^a^	Cutoff threshold of probability = .1								Cutoff threshold of probability = .5							
	MC	DIF	LLM	SAME	Accuracy (%)	Precision (%)	Recall (%)	F1-score	MC	DIF	LLM	SAME	Accuracy (%)	Precision (%)	Recall (%)	F1-score
Aortic regurgitation	0	0	3	101	97.12	97.12	100.00	0.99	2	0	3	99	95.19	97.06	98.02	0.98
Aortic stenosis	1	1	2	78	95.12	96.30	97.50	0.97	2	0	2	78	95.12	97.50	97.50	0.98
Diastolic dysfunction	2	1	1	91	95.79	97.85	96.81	0.97	2	1	1	91	95.79	97.85	96.81	0.97
LAE	2	0	2	159	97.55	98.76	98.76	0.99	2	0	2	159	97.55	98.76	98.76	0.99
LV size	5	3	2	190	95.00	97.44	95.96	0.97	5	3	2	190	95.00	97.44	95.96	0.97
LVEF	1	1	1	279	98.94	99.29	99.29	0.99	2	1	1	278	98.58	99.29	98.93	0.99
LVH	2	1	2	151	96.79	98.05	98.05	0.98	2	1	2	151	96.79	98.05	98.05	0.98
Mitral regurgitation	1	1	1	134	97.81	98.53	98.53	0.99	4	0	1	132	96.35	99.25	97.06	0.98
Mitral stenosis	2	1	1	17	80.95	89.47	85.00	0.87	2	1	1	17	80.95	89.47	85.00	0.87
Pericardial effusion	2	1	3	186	96.88	97.89	98.41	0.98	2	1	3	186	96.88	97.89	98.41	0.98
Prosthetic aortic valve	2	0	0	29	93.55	100.00	93.55	0.97	2	0	0	29	93.55	100.00	93.55	0.97
Prosthetic mitral valve	2	0	0	4	66.67	100.00	66.67	0.80	2	0	0	4	66.67	100.00	66.67	0.80
Pulmonic HTN	0	0	0	15	100.00	100.00	100.00	1.00	0	0	0	15	100.00	100.00	100.00	1.00
Pulmonic regurgitation	0	0	2	26	92.86	92.86	100.00	0.96	1	0	2	25	89.29	92.59	96.15	0.94
Pulmonic stenosis	1	0	0	1	50.00	100.00	50.00	0.67	1	0	0	1	50.00	100.00	50.00	0.67
RA pressure	6	0	2	50	86.21	96.15	89.29	0.93	6	0	2	50	86.21	96.15	89.29	0.93
RAE	2	0	2	103	96.26	98.10	98.10	0.98	2	0	2	103	96.26	98.10	98.10	0.98
RV function	4	1	1	87	93.55	97.75	94.57	0.96	5	1	1	86	92.47	97.73	93.48	0.96
RV size	3	0	8	91	89.22	91.92	96.81	0.94	3	0	7	91	90.10	92.86	96.81	0.95
RVSP	0	2	2	142	97.26	97.26	98.61	0.98	0	2	2	142	97.26	97.26	98.61	0.98
Tricuspid regurgitation	1	0	0	139	99.29	100.00	99.29	1.00	2	0	0	138	98.57	100.00	98.57	0.99
WMA	7	0	3	130	92.86	97.74	94.89	0.96	7	0	3	130	92.86	97.74	94.89	0.96
*All*	46	13	38	2203	95.78	97.74	97.39	0.98	56	11	37	2195	95.48	97.86	97.04	0.97

The model was trained on 2986 reports with sequence length = 512, batch size = 4, and epochs = 8.

Abbreviations: DIF, chart review result and language model result are different; LLM, large language model result only; MC, manually confirmed only; SAME, chart review result and language model result are same; LVEF, left ventricular ejection fraction; LAE, left atrial enlargement; LVH, left ventricular hypertrophy. RAE, right atrial enlargement; RVSP, right ventricular systolic pressure; WMA, wall motion abnormalities.

a The performance of prosthetic tricuspid valve, prosthetic pulmonic valve, and tricuspid stenosis was not evaluated because of no confirmed cases.

**Table 2.** The performance of the fine-tuned BART-Large-CNN model against the manual annotated results in the echocardiography report validation dataset (*n* = 300) at 0.1 and 0.5 cutoff thresholds of probability.

Extracted outcome^a^	Cutoff threshold of probability = .1								Cutoff threshold of probability = .5							
	MC	DIF	LLM	SAME	Accuracy (%)	Precision (%)	Recall (%)	F1-score	MC	DIF	LLM	SAME	Accuracy (%)	Precision (%)	Recall (%)	F1-score
Aortic regurgitation	0	0	3	101	97.12	97.12	100.00	0.99	0	0	3	101	97.12	97.12	100.00	0.99
Aortic stenosis	0	0	2	80	97.56	97.56	100.00	0.99	1	0	2	79	96.34	97.53	98.75	0.98
Diastolic dysfunction	2	1	1	91	95.79	97.85	96.81	0.97	2	1	1	91	95.79	97.85	96.81	0.97
LAE	2	0	3	159	96.95	98.15	98.76	0.98	2	0	3	159	96.95	98.15	98.76	0.98
LV size	5	1	1	192	96.48	98.97	96.97	0.98	5	1	1	192	96.48	98.97	96.97	0.98
LVEF	1	2	1	278	98.58	98.93	98.93	0.99	1	2	1	278	98.58	98.93	98.93	0.99
LVH	1	0	2	153	98.08	98.71	99.35	0.99	3	1	2	150	96.15	98.04	97.40	0.98
Mitral regurgitation	2	0	1	134	97.81	99.26	98.53	0.99	2	0	1	134	97.81	99.26	98.53	0.99
Mitral stenosis	0	0	1	20	95.24	95.24	100.00	0.98	0	0	1	20	95.24	95.24	100.00	0.98
Pericardial effusion	4	1	2	184	96.34	98.40	97.35	0.98	4	1	1	184	96.84	98.92	97.35	0.98
Prosthetic aortic valve	4	0	1	27	84.38	96.43	87.10	0.92	4	0	1	27	84.38	96.43	87.10	0.92
Prosthetic mitral valve	2	0	0	4	66.67	100.00	66.67	0.80	2	0	0	4	66.67	100.00	66.67	0.80
Pulmonic HTN	1	0	0	14	93.33	100.00	93.33	0.97	1	0	0	14	93.33	100.00	93.33	0.97
Pulmonic regurgitation	0	0	1	26	96.30	96.30	100.00	0.98	0	0	1	26	96.30	96.30	100.00	0.98
Pulmonic stenosis	1	0	0	1	50.00	100.00	50.00	0.67	2	0	0	0	0.00	0.00	0.00	0.00
RA pressure	7	1	2	48	82.76	94.12	85.71	0.90	8	0	2	48	82.76	96.00	85.71	0.91
RAE	2	0	1	103	97.17	99.04	98.10	0.99	2	0	1	103	97.17	99.04	98.10	0.99
RV function	4	1	1	87	93.55	97.75	94.57	0.96	5	1	1	86	92.47	97.73	93.48	0.96
RV size	5	0	8	89	87.25	91.75	94.68	0.93	6	0	8	88	86.27	91.67	93.62	0.93
RVSP	1	1	3	142	96.60	97.26	98.61	0.98	1	1	3	142	96.60	97.26	98.61	0.98
Tricuspid regurgitation	1	1	1	138	97.87	98.57	98.57	0.99	2	0	1	138	97.87	99.28	98.57	0.99
WMA	9	0	4	128	90.78	96.97	93.43	0.95	9	0	4	128	90.78	96.97	93.43	0.95
*All*	54	9	39	2199	95.57	97.86	97.21	0.98	62	8	38	2192	95.30	97.94	96.91	0.97

The model was trained on 2986 reports with sequence length = 512, batch size = 4, and epochs = 8.

Abbreviations: DIF, chart review result and language model result are different; LLM, large language model result only; MC, manually confirmed only; SAME, chart review result and language model result are same; LVEF, left ventricular ejection fraction; LAE, left atrial enlargement; LVH, left ventricular hypertrophy. RAE, right atrial enlargement; RVSP, right ventricular systolic pressure; WMA, wall motion abnormalities.

a The performance of prosthetic tricuspid valve, prosthetic pulmonic valve, and tricuspid stenosis was not evaluated because of no confirmed cases.

Tables 3 and 4 summarize the performance of fine-tuned BioclinicalBERT and BART-Large-CNN at rand seed of 42 on the validation dataset at the probability thresholds of 0.1 and 0.5 for cardiac catheterization reports. Overall, BART-Large-CNN slightly outperformed BioclinicalBERT (accuracy 95.21% vs 94.16%; precision 97.24% vs 96.38%; recall 96.02% vs 95.34%; F1-score 0.97 vs 0.96 at the probability threshold = 0.1 and accuracy 95.09% vs 93.76%; precision 97.23% vs 96.47%; recall 95.90% vs 94.88%; F1-score 0.97 vs 0.96 at the probability threshold = 0.5). For each labeled category, most of them also achieved high performance by both fine-tuned models (accuracy, precision and recall over 90% and F1-score >0.9) except for some categories, including diagonal 2 (accuracy 89.06% for both models), diagonal 3 (accuracy 75.00%, recall 80.00%, F1-score 0.86 for BioclinicalBERT and accuracy 73.33%, recall 73.33%, F1-score 0.85 for BART-Large-CNN), OM2 (accuracy 75.47%, precision 78.43%, F1-score 0.84 at threshold of 0.1 and accuracy 75.58%, precision 78.00%, recall 88.64%, F1-score 0.83 at threshold of 0.5 for BioclinicalBERT), OM4 (accuracy 50.00%, precision 50.00%, F1-score 0.67 at both thresholds 0.1 and 0.5 for BART-Large-CNN), and OM5 (accuracy 50.00%, precision 50.00%, F1-score 0.67 at both thresholds 0.1 and 0.5 for BART-Large-CNN). Similar performance of the 2 models at the other rand seeds are presented in Table S7. The mean and SD of the performance across the 5 runs are summarized in Table S8. The BART-Large-CNN model slightly outperformed the BioclinicalBERT model with mean accuracy 94.9% vs 94.3%; precision 96.7% vs 96.3%; recall 96.1% vs 95.7%, and F1-score 0.96 vs 0.96 at the probability threshold of 0.1, and 94.9% vs 93.7%; precision 96.8% vs 96.5%; recall 96.0% vs 95.0%, and F1-score 0.96 vs 0.96 at the probability threshold of 0.5.

**Table 3.** The performance of the fine-tuned BioclinicalBERT model against the manual annotated results in the cardiac catheterization report validation dataset (*n* = 300) at 0.1 and 0.5 cutoff thresholds of probability.

Extracted outcome^a^	Cutoff threshold of probability = .1								Cutoff threshold of probability = .5							
	MC	DIF	LLM	SAME	Accuracy (%)	Precision (%)	Recall (%)	F1-score	MC	DIF	LLM	SAME	Accuracy (%)	Precision (%)	Recall (%)	F1-score
Diagonal 1	7	4	2	118	90.08	95.16	91.47	0.93	8	4	2	117	89.31	95.12	90.70	0.93
Diagonal 2	4	1	2	57	89.06	95.00	91.94	0.93	5	0	2	57	89.06	96.61	91.94	0.94
Diagonal 3	3	0	1	12	75.00	92.31	80.00	0.86	3	0	1	12	75.00	92.31	80.00	0.86
LAD	5	12	0	272	94.12	95.77	94.12	0.95	7	12	0	270	93.43	95.74	93.43	0.95
LCX	2	9	5	252	94.03	94.74	95.82	0.95	3	9	4	251	94.01	95.08	95.44	0.95
LIMA-LAD	0	0	0	3	100.00	100.00	100.00	1.00	0	0	0	3	100.00	100.00	100.00	1.00
Left main	0	2	0	283	99.30	99.30	99.30	0.99	0	2	0	283	99.30	99.30	99.30	0.99
OM1	7	3	0	116	92.06	97.48	92.06	0.95	7	3	0	116	92.06	97.48	92.06	0.95
OM2	2	2	9	40	75.47	78.43	90.91	0.84	3	2	9	39	73.58	78.00	88.64	0.83
OM3	0	0	0	22	100.00	100.00	100.00	1.00	0	0	0	22	100.00	100.00	100.00	1.00
OM4	0	0	0	1	100.00	100.00	100.00	1.00	0	0	0	1	100.00	100.00	100.00	1.00
OM5	0	0	0	1	100.00	100.00	100.00	1.00	0	0	0	1	100.00	100.00	100.00	1.00
PDA	5	0	3	98	92.45	97.03	95.15	0.96	5	0	3	98	92.45	97.03	95.15	0.96
PLV	1	1	0	89	97.80	98.89	97.80	0.98	1	1	0	89	97.80	98.89	97.80	0.98
RCA	4	7	0	266	96.03	97.44	96.03	0.97	6	7	0	264	95.31	97.42	95.31	0.96
Ramus	1	0	0	42	97.67	100.00	97.67	0.99	1	0	0	42	97.67	100.00	97.67	0.99
SVG-D1	0	0	0	1	100.00	100.00	100.00	1.00	0	0	0	1	100.00	100.00	100.00	1.00
SVG-OM1	0	0	0	2	100.00	100.00	100.00	1.00	1	0	0	1	50.00	100.00	50.00	0.67
SVG-PDA	0	0	0	1	100.00	100.00	100.00	1.00	0	0	0	1	100.00	100.00	100.00	1.00
*ALL*	41	41	22	1676	94.16	96.38	95.34	0.96	50	40	21	1668	93.76	96.47	94.88	0.96

The model was trained on 1584 reports with sequence length = 512, batch size = 4, and epochs = 8.

Abbreviations: DIF, chart review result and language model result are different; LLM, large language model result only; MC, manually confirmed only; SAME, chart review result and language model result are same; LAD, left anterior descending artery; LCX, left circumflex coronary artery; LIMA, left internal mammary artery ; OM1, obtuse marginal 1; OM2, obtuse marginal 2; OM3, obtuse marginal 3; OM4, obtuse marginal 4; OM5, obtuse marginal 5; PDA, posterior descending artery; PLV, posterior left ventricular artery; RCA, right coronary artery; SVG, saphenous vein graft; D1, Diagonal 1.

a The performance of the outcome categories in Table S2, but not listed here was not evaluated because of no confirmed cases.

**Table 4:** The performance of the fine-tuned BART-Large-CNN model against the manual annotated results in the cardiac catheterization report validation dataset (*n* = 300) at 0.1 and 0.5 cutoff thresholds of probability.

Extracted outcome^a^	Cutoff threshold of probability = .1								Cutoff threshold of probability = .5							
	MC	DIF	LLM	SAME	Accuracy (%)	Precision (%)	Recall (%)	F1-score	MC	DIF	LLM	SAME	Accuracy (%)	Precision (%)	Recall (%)	F1-score
Diagonal 1	6	6	1	117	90.00	94.35	90.70	0.92	6	6	1	117	90.00	94.35	90.70	0.92
Diagonal 2	4	1	2	57	89.06	95.00	91.94	0.93	4	1	2	57	89.06	95.00	91.94	0.93
Diagonal 3	4	0	0	11	73.33	100.00	73.33	0.85	4	0	0	11	73.33	100.00	73.33	0.85
LAD	3	9	0	277	95.85	96.85	95.85	0.96	4	9	0	276	95.50	96.84	95.50	0.96
LCX	0	6	5	257	95.90	95.90	97.72	0.97	0	6	5	257	95.90	95.90	97.72	0.97
LIMA-LAD	0	0	0	3	100.00	100.00	100.00	1.00	0	0	0	3	100.00	100.00	100.00	1.00
Left main	0	1	0	284	99.65	99.65	99.65	1.00	0	1	0	284	99.65	99.65	99.65	1.00
OM1	9	1	0	116	92.06	99.15	92.06	0.95	10	1	0	115	91.27	99.14	91.27	0.95
OM2	0	0	2	44	95.65	95.65	100.00	0.98	0	0	2	44	95.65	95.65	100.00	0.98
OM3	0	0	0	22	100.00	100.00	100.00	1.00	0	0	0	22	100.00	100.00	100.00	1.00
OM4	0	0	1	1	50.00	50.00	100.00	0.67	0	0	1	1	50.00	50.00	100.00	0.67
OM5	0	0	1	1	50.00	50.00	100.00	0.67	0	0	1	1	50.00	50.00	100.00	0.67
PDA	9	0	1	94	90.38	98.95	91.26	0.95	9	0	1	94	90.38	98.95	91.26	0.95
PLV	1	2	1	88	95.65	96.70	96.70	0.97	1	2	1	88	95.65	96.70	96.70	0.97
RCA	0	7	1	270	97.12	97.12	97.47	0.97	0	7	1	270	97.12	97.12	97.47	0.97
Ramus	1	0	0	42	97.67	100.00	97.67	0.99	1	0	0	42	97.67	100.00	97.67	0.99
SVG-D1	0	0	0	1	100.00	100.00	100.00	1.00	0	0	0	1	100.00	100.00	100.00	1.00
SVG-OM1	0	0	0	2	100.00	100.00	100.00	1.00	0	0	0	2	100.00	100.00	100.00	1.00
SVG-PDA	0	0	0	1	100.00	100.00	100.00	1.00	0	0	0	1	100.00	100.00	100.00	1.00
*ALL*	37	33	15	1688	95.21	97.24	96.02	0.97	39	33	15	1686	95.09	97.23	95.90	0.97

The model was trained on 1584 reports with sequence length = 512, batch size = 4, and epochs = 8.

Abbreviations: DIF, chart review result and language model result are different; LLM, large language model result only; MC, manually confirmed only; SAME, chart review result and language model result are same; LAD, left anterior descending artery; LCX, left circumflex coronary artery; LIMA, left internal mammary artery ; OM1, obtuse marginal 1; OM2, obtuse marginal 2; OM3, obtuse marginal 3; OM4, obtuse marginal 4; OM5, obtuse marginal 5; PDA, posterior descending artery; PLV, posterior left ventricular artery; RCA, right coronary artery; SVG, saphenous vein graft; D1, Diagonal 1.

a The performance of the outcome categories in Table S2, but not listed here was not evaluated because of no confirmed cases.

### Model performance as a function of training set size

**Figure 2.**
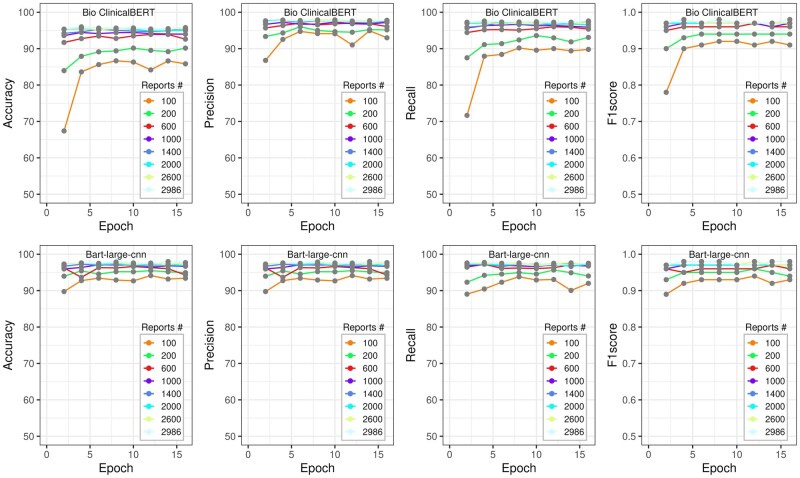
The performance metrics (accuracy, precision, recall, and F1-score) vs the number of training epochs and the total number of training reports for echocardiography. The BioclinicalBERT model result is presented at the top, while the result from the BART-Large-CNN model at the bottom.

**Figure 3.**
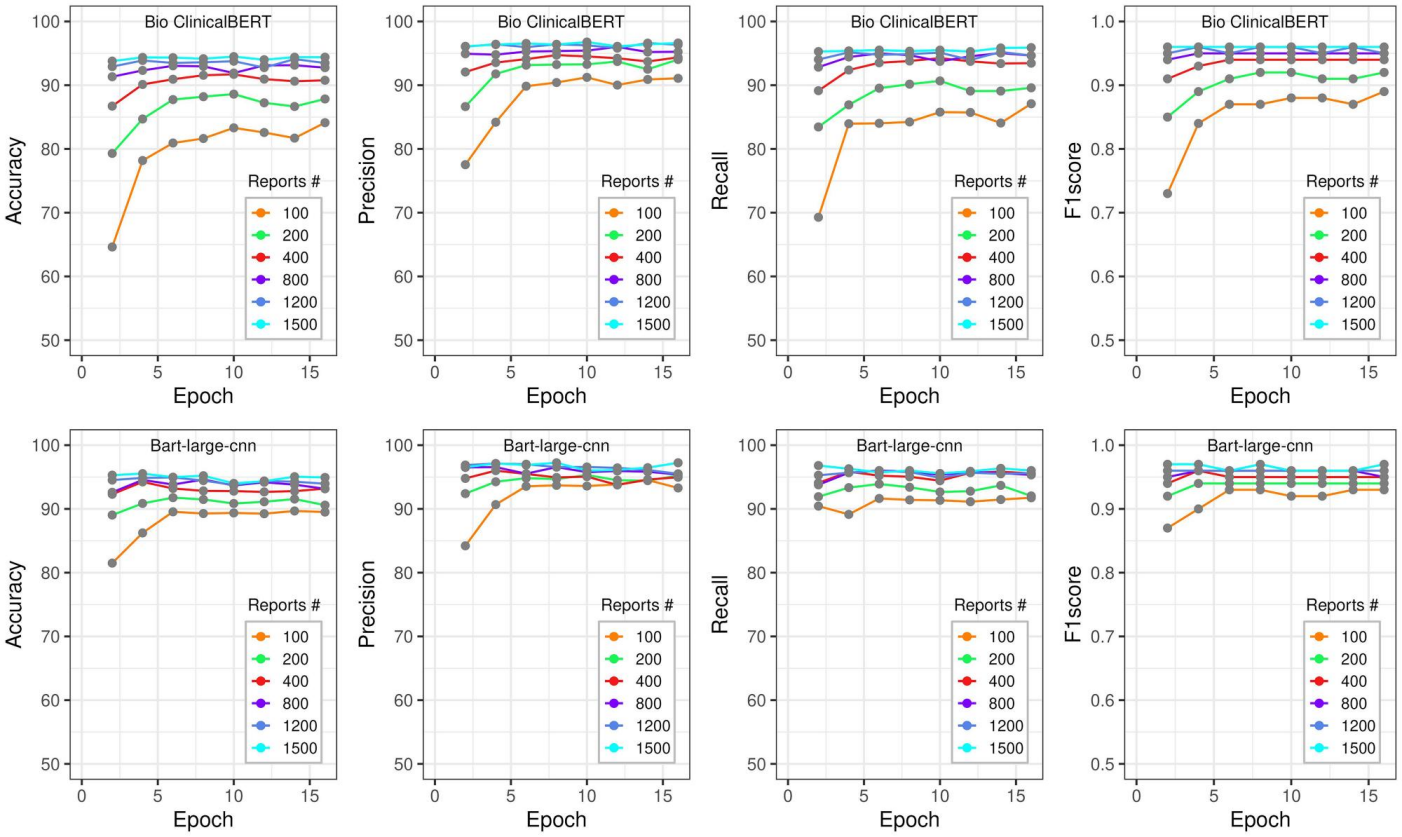
The performance metrics (accuracy, precision, recall, and F1-score) vs the number of training epochs and the total number of training reports for cardiac catheterization. The BioclinicalBERT model result is presented at the top, while the result from the BART-Large-CNN model at the bottom.

The relationship between model performance at a probability threshold of 0.1 vs the number of training reports and training epochs is shown in Figure 2 for echocardiography reports and Figure 3 for cardiac catheterization reports, respectively. Overall, the BART-Large-CNN model was less sensitive to the number of both training reports and training epochs. The model’s performance increased with the number of training reports but reached a plateau of around 1000 training reports for both report groups and models. Each model achieved stable performance at 8 training epochs regardless of the report group, models, and the total number of training reports.

### Discrepancy analysis

Discrepancies between the fine-tuned models and the manually annotated reference data in the validation datasets at the probability threshold of 0.1 were systematically analyzed and summarized in Table S9. Several common error patterns were identified: (1) the models extracted completely different values compared to the manual annotated values (13 cases from the BioclinicalBERT model and 9 cases from the BART-Large-CNN model for echocardiography; 41 cases from the BioClinicalBERT model and 33 cases from the BART-Large-CNN model for cardiac catheterization), (2) the models generated hallucinated values in some categories that were not mentioned in the annotated reports (38 cases from the BioclinicalBERT model and 39 cases from the BART-Large-CNN model for echocardiography; 22 cases from the BioClinicalBERT model and 15 from the BART-Large-CNN model for cardiac catheterization), (3) the models extracted the same values as manual annotation, but failed to correctly label them due to low confidence scores below the 0.1 threshold (20 cases from the BioclinicalBERT model and 20 cases from the BART-Large-CNN model for echocardiography; 14 cases from the BioClinicalBERT model and 11 cases from the BART-Large-CNN model for cardiac catheterization), and (4) the model extracted completely different values compared to the manual annotated values with low probability scores <0.1 (26 cases from the BioclinicalBERT model and 34 cases from the BART-Large-CNN model for echocardiography; 27 cases from the BioClinicalBERT model and 26 cases from BART-Large-CNN model for cardiac catheterization).

## Discussion

In this study, we successfully implemented and fine-tuned 2 open-source transformer-based LMs, BioClinicalBERT and BART-Large-CNN, to convert free-text echocardiography and cardiac catheterization reports into structured data with high accuracy. For example, both fine-tuned BioclinicalBERT and the BART-Large-CNN models were able to correctly extract “60%” as the value of LVEF and “mild” as the value of aortic regurgitation from the echo text “1. Normal left ventricular systolic function, EF 60% 2. Mild aortic valve regurgitation,” and “normal” as the value of left main, “minimal luminal irregularities” as the values of LAD, LCX, and RCA, “ostial 50% stenosis” as the value of diagonal 2 from the cardiac catheterization text “Left Main Artery is normal. Left Anterior Descending Artery has minimal luminal irregularities. Diagonal 2 has ostial 50% stenosis. Circumflex Artery is large and has minimal luminal irregularities. Right Coronary Artery is large and has minimal luminal irregularities.” To our knowledge, this is the first study to systematically extract a broad range of clinically relevant information from both echocardiography and cardiac catheterization reports using locally deployed, fine-tuned LLMs. Notably, BART-Large-CNN, despite not being pretrained on medical text, performed comparably to BioClinicalBERT, demonstrating that general-domain transformer models can be effectively adapted for highly specialized medical text extraction tasks. Both models achieved strong overall performance, with accuracy, precision, recall, and F1-scores exceeding 90% across report types regardless of the rand seed selection. In addition, model performance increased with the number of annotated training reports but plateaued at approximately 1000 reports, indicating that robust performance can be achieved with a relatively limited annotation burden. Together, these findings underscore the feasibility of utilizing locally deployed LMs to extract a comprehensive set of structured information from unstructured clinical text.

Although the fine-tuned models in our study demonstrated strong performance, several recurring types of discrepancies were observed. First, the models generated information different from the values described in the reports. For example, the models incorrectly extracted the historic LVEF value of 60%-65% rather than 35% as the LEVF value of current study from the echo text “The earlier study shows LVEF estimated at 60-65%. Current study shows estimated LVEF 35%, and thus is significantly lower.” Second, the models generated the values not described in the reports. For instance, the models correctly extracted “mild disease” as the value of diagonal 1 and “mild ostial disease” for diagonal 3, but also incorrectly extracted “mild ostial disease” for diagonal 2, which was not mentioned, from the cardiac catheterization text “1st diagonal is medium in caliber and has mild disease in mid segment. 2nd diagonal is very small in caliber. 3rd diagonal is small in caliber and has mild ostial disease.” Third, the models failed to extract the values described in the reports or exclude retrieved the values described due to the probability score less than the cutoff threshold. For example, the models extracted “minimal” as the value of aortic stenosis with an almost zero (2.0e-18) probability score from the echo text “Valve findings: Mild significant Valvular diseases. TR, MR minimal AS.” Fourth, the stepwise reduction in performance from diagonal 1 to diagonal 3 in cardiac catheterization reports likely reflects the anatomical similarity and decreasing frequency of these categories described in the reports. Therefore, incorporating additional measures, such as postprocessing, threshold adjustment, rule-based safeguards or manual monitoring and reviews of samples of transformer-based LM generated results, can help mitigate these discrepancies in future work for the transformer-based LMs implementation. For instance, hallucinated values generated by the transformer-based models may be minimized by first detecting the presence of relevant categories and then calibrating probability thresholds to suppress outputs for categories that are not explicitly mentioned.

Consistent with prior studies,^19^ our study demonstrates that an open-source model deployed on a local server can accurately extract structured data from unstructured clinical text. This offline approach offers significant advancement over earlier studies that relied on externally hosted or closed-source LLMs accessed via data transfer and proprietary application programming interfaces.^25^^,^^26^ By eliminating the need to transmit sensitive data outside institutional boundaries, this strategy better addresses privacy and security concerns, supporting scalable and compliant deployment within health-care settings.

Large language model performance typically improves with increasing volumes of training data, although this relationship is not strictly linear.^27^ Optimizing performance, therefore, requires a careful tradeoff between data quantity, quality, model size, and computational budget. In clinical applications, model training is most often constrained by the availability of high-quality annotated data, as annotation typically requires substantial clinician time, which is both costly and limited in availability. Our study showed that increasing the number of annotated echocardiography and cardiac catheterization reports can boost transformer-based LM performance. However, performance plateaued at approximately 1000 reports. Adding training data beyond 1000 did not result in significant improvements in model performance. Although data sufficiency thresholds are task- and model-dependent and may vary across clinical domains and LLM architectures, a similar plateau was observed for both echocardiography and cardiac catheterization reports in our study, despite these representing different types of clinical documentation.

One advantage of the pretrained generative LLMs is their ability to extract information in a zero-shot learning with variable accuracy.^11–19^ However, in our study, neither the BioclinicalBERT nor the BART-Large-CNN model was able to extract meaningful information using zero-shot inference without further fine-tuning. As shown in Tables S10 and S11, the zero-shot learning outputs for a small set of reports assigned all labeled categories to a single text string that was unrelated to the true category descriptions and associated with a near-zero probability score. Such inability was likely due to the specialized nature and complexity of the highly structured, measurement-heavy clinical reports. Therefore, the fine-tuning step is a crucial step for accurately and reliably extracting information from these highly specialized clinical report types within our modeling framework. As shown in Tables S10 and S11, the outputs generated by the fine-tuned models were associated with a near-one probability score and consistent with annotated results for these categories described in the reports. The fine-tuned models also generated noise outputs for the categories not described in the reports, but these outputs are automatically ignored because the corresponding near-zero probability score was less than the cutoff threshold. Although our research focused on extracting data from echocardiography and cardiac catheterization reports, the proposed framework is flexible and readily extensible to other forms of unstructured health-care data, such as radiology reports, pathology reports, and clinical progress notes, enabling the creation of large-scale databases for clinical research and downstream AI developments.

Recently developed large-scale LLMs can manage and solve more complex tasks via advanced techniques, such as reasoning skills.^28^ Implementing such large-scale LLMs typically requires high-performance, dedicated servers that include significant GPU power (multiple high-VRAM GPUs), large system memory, fast disk storage, and powerful CPUs. These resource requirements limit our ability to evaluate large-scale LLMs in the current study systematically. As our computing environment expands, future work will explore the application and potential advantages of larger models for structured information extraction from clinical text.

Our study has successfully demonstrated the feasibility of locally fine-tuned transformer-based LMs for extracting structured data from unstructured clinical reports. Although the fine-tuning process of these transformer-based LMs is relatively resource-intensive, inference and downstream deployment require substantially fewer resources. Practical operationalization of these fine-tuned transformer-based LMs can follow several pathways. For example, batch extraction workflows can be implemented to periodically process large volumes of historical reports, facilitating retrospective research and the creation of specialized databases. Alternatively, real-time integration into EHR systems could enable the immediate structuring of newly generated reports, supporting timely clinical decision-making. Furthermore, the extracted structured data can be seamlessly integrated into research pipelines, facilitating various downstream research needs. These operational models would leverage the robust performance and privacy advantages of locally deployed LLMs, enhancing both research efficiency and clinical utility.

It is worth acknowledging the growing availability of commercial enterprise LLM deployments at many health-care systems and academic institutions. Institutionally hosted enterprise models can also perform well on structured extraction and similar downstream tasks via zero-shot learning or fine-tuning from a smaller dataset, reducing development time and operational overhead. For institutions with access to these platforms and appropriate governance frameworks, such solutions represent another practical and efficient alternative. However, reliance on enterprise models introduces considerations around data control, customization limits, vendor dependency, and long-term cost. In contrast, the open-source, locally deployed approach described here prioritizes full data sovereignty, transparency, and adaptability, and may be better aligned with institutions that require tight control over infrastructure or wish to extend the pipeline beyond the capabilities of commercial offerings. Presenting both options clarifies the decision space for readers and highlights that the optimal choice depends on institutional resources, regulatory context, and project goals.

The structured outputs generated by these transformer-based LMs recorded verbatim as they appeared in the original reports and appear largely amenable to de-identification, suggesting that the annotated reports could be potentially shared with the broader community to enable benchmarking and collaborative development of more generalizable extraction tools. Beyond text-based modeling, these curated datasets also provide a valuable foundation for future work exploring whether deep learning approaches can derive selected measurements directly from raw imaging data (DICOM), offering a complementary image-to-structured-data pathway that could further automate and validate clinical data capture. Furthermore, the verbatim text captured by these transformer-based LMs offers downstream applications greater flexibility in defining more granular need categories.

Our study has several limitations. First, this study relied on a single fixed question for each target label, and performance under alternative clinically plausible phrasings was not evaluated, limiting assessment of prompt robustness. Future work should systematically examine prompt variation to quantify sensitivity to wording and to identify formulations that optimize extraction performance. Second, model optimization focused primarily on the number of training epochs. Tuning additional hyperparameters, including learning rate, maximal sequence length, batch size, and weight decay ratio, could further boost the performance.^29^ Third, we only explored the performance at the 2 selected probability score thresholds of 0.1 and 0.5; a more systematic threshold-sweep analysis across the full range [0,1] could achieve improved optimization and better calibration in future work. Fourth, although all predefined study categories were included during the fine-tuning process, the performance of some categories in both echocardiography and cardiac catheterization reports could not be fully assessed due to the absence of corresponding cases in the validation datasets. In addition, some categories (eg, pulmonic stenosis, prosthetic mitral valve, OM4, OM5, SVG-D1, SVG-OM1, and SVG-PDA) required cautious interpretation because of small sample sizes. Larger datasets containing more examples of these rare categories will be necessary to improve robustness and reproducibility. Fifth, the echocardiography reports were split for training and validation at report level rather than patient level. Such splitting may allow reports from the same patient to appear in both training and validation sets and thus introduce a potential risk of data leakage and performance inflation. Future studies will mitigate this by enforcing patient-level splits to more conservatively assess generalization. Sixth, our study did not assess interrater agreement and reliance on a single annotator in the study may introduce subjective or systematic bias into the reference annotations. Incorporating multiple annotators and interrater agreement in future work would strengthen the reliability of the ground truth. Seventh, the model training and evaluation in this study were conducted using a single random seed rather than repeated runs, which may limit assessment of performance variability and robustness. Lastly, while the fine-tuned models performed well within the KPSC health-care system, external validation in future studies will be necessary to strengthen robustness and generalizability, as well as to assess real-world performance applied in other health-care settings, which may vary due to differences in report templates, dictation styles, and setting conventions.

## Conclusion

This study demonstrates the feasibility of using fine-tuned transformer-based LMs to extract data from unstructured echocardiography and cardiac catheterization reports effectively. A similar framework can be applied to other types of unstructured health-care data. The approach highlighted the potential of locally deployed transformer-based LMs to support automatic information extraction from unstructured medical data, enhancing clinical research efficiency and enabling downstream clinical and analytic applications.

## Supplementary Material

ooag036_Supplementary_Data

## Data Availability

The data underlying this article were extracted from electronic health records and cannot be shared publicly for the privacy of individuals who participated in the study.

## References

[1] Otto CM , Nishimura RA, Bonow RO, et al C. 2020 ACC/AHA guideline for the management of patients with valvular heart disease: executive summary: a report of the American College of Cardiology/American Heart Association Joint Committee on Clinical Practice Guidelines. Circulation. 2021;143:e35-e71. 10.1161/CIR.0000000000000932 33332149

[2] Mitchell C , Rahko PS, Blauwet LA, et al Guidelines for performing a comprehensive transthoracic echocardiographic examination in adults: recommendations from the American Society of Echocardiography. J Am Soc Echocardiogr. 2019;32:1-64. 10.1016/j.echo.2018.06.004 30282592

[3] Rao SV , O’Donoghue ML, Ruel M, et al 2025 ACC/AHA/ACEP/NAEMSP/SCAI guideline for the management of patients with acute coronary syndromes: a report of the American College of Cardiology/American Heart Association Joint Committee on Clinical Practice Guidelines. Circulation. 2025;151:e771-e862. 10.1161/CIR.0000000000001309 40014670

[4] Bangalore S , Fearon WF, Fugar S, et al; American Heart Association Interventional Care Committee of the Council on Clinical Cardiology; and Council on Cardiovascular and Stroke Nursing. Evidence-based practices in the cardiac catheterization laboratory: invasive epicardial coronary physiologic assessment: a scientific statement from the American Heart Association. Circulation. 2025;153:e25-e41. 10.1161/CIR.0000000000001389 41263068

[5] Friedman C , Alderson PO, Austin JH, Cimino JJ, Johnson SB. A general natural-language text processor for clinical radiology. J Am Med Inform Assoc. 1994;1:161-174. 10.1136/jamia.1994.95236146 7719797 PMC116194

[6] Smajić A , Karlović R, Bobanović Dasko M, Lorencin I. Large language models for structured and semi-structured data, recommender systems and knowledge base engineering: a survey of recent techniques and architectures. Electronics (Basel). 2025;14:3153. 10.3390/electronics14153153.

[7] Solomon MD , Tabada G, Allen A, Sung SH, Go AS. Large-scale identification of aortic stenosis and its severity using natural language processing on electronic health records. Cardiovasc Digit Health J. 2021;2:156-163. 10.1016/j.cvdhj.2021.03.003 35265904 PMC8890044

[8] Nath C , Albaghdadi MS, Jonnalagadda SR. A natural language processing tool for large-scale data extraction from echocardiography reports. PLoS One. 2016;11:e0153749. 10.1371/journal.pone.0153749 27124000 PMC4849652

[9] Dong T , Sunderland N, Nightingale A, et al Development and evaluation of a natural language processing system for curating a trans-thoracic echocardiography (TTE) database. Bioengineering (Basel). 2023;10:1307. 10.3390/bioengineering10111307 38002431 PMC10669818

[10] Xie F , Lee MS, Allahwerdy S, Getahun D, Wessler B, Chen W. Identifying the severity of heart valve stenosis and regurgitation among a diverse population within an integrated health care system: natural language processing approach. JMIR Cardio. 2024;8:e60503. 10.2196/60503 39348175 PMC11474122

[11] Barak-Corren Y , Gupta M, Tang J, et al From text to data: automatically extracting data from catheterization reports using generative artificial intelligence. J Soc Cardiovasc Angiogr Interv. 2025;4:102242. 10.1016/j.jscai.2024.102242 40230663 PMC11993886

[12] Burford KG , Itzkowitz NG, Ortega AG, Teitler JO, Rundle AG. Use of generative AI to identify helmet status among patients with micromobility-related injuries from unstructured clinical notes. JAMA Netw Open. 2024;7:e2425981. 10.1001/jamanetworkopen.2024.25981 39136946 PMC11322845

[13] Lee D , Vaid A, Menon KM, Freeman R, Matteson DS, Marin ML, et al Using large language models to automate data extraction from surgical pathology report: retrospective cohort study. JMIR Form Res 2025;9:e64544. 10.2196/64544 40194317 PMC11996145

[14] Chen D , Alnassar SA, Avison KE, Huang RS, Raman S. Large language model applications for health information extraction in oncology: scoping review. JMIR Cancer 2025;11:e65984. 10.2196/65984 40153782 PMC11970800

[15] Alkhalaf M , Yu P, Yin M, Deng C. Applying generative AI with retrieval augmented generation to summarize and extract key clinical information from electronic health records. J Biomed Inform. 2024;156:104662. 38880236 10.1016/j.jbi.2024.104662

[16] Kim MS , Chung P, Aghaeepour N, Kim N. Information extraction from clinical texts with generative pre-trained transformer models. Int J Med Sci. 2025; 22:1015-1028. 10.7150/ijms.103332 40027192 PMC11866537

[17] Goel A , Gueta A, Gilon O, et al LLMs accelerate annotation for medical information extraction. In: *Machine Learning for Health (ML4H)*. PMLR; 2023:82-100.

[18] Fornasiere R , Brunello N, Scotti V, Carman M. Medical information extraction with large language models. In: *Proceedings of the 7th International Conference on Natural Language and Speech Processing (ICNLSP 2024)*. Association for Computational Linguistics; 2024: 456-466.

[19] Dao N , Quesada L, Hassan SM, et al Generative artificial intelligence for automated data extraction from unstructured medical text. JAMIA Open. 2025;8:ooaf097. 10.1093/jamiaopen/ooaf097 40918939 PMC12410982

[20] Alsentzer E , Murphy JR, Boag W, et al 2019. Publicly available clinical BERT embeddings. arXiv, arXiv:1904.03323, preprint: not peer reviewed.

[21] Lewis M , Liu Y, Goyal N, et al BART: Denoising sequence-to-sequence pre-training for natural language generation, translation, and comprehension. arXiv, arXiv:1910.13461, 2019, preprint: not peer reviewed.

[22] Tam TYC , Sivarajkumar S, Kapoor S, et al A framework for human evaluation of large language models in healthcare derived from literature review. NPJ Digit Med. 2024;7:258. 39333376 10.1038/s41746-024-01258-7 PMC11437138

[23] Facebook/bart-large-cnn. Hugging Face. https://huggingface.co/facebook/bart-large-cnn

[24] Emilyalsentzer/Bio_ClinicalBERT. Hugging Face. Accessed July 12, 2025. https://huggingface.co/emilyalsentzer/Bio_ClinicalBERT

[25] Sushil M , Kennedy VE, Mandair D, Miao BY, Zack T, Butte AJ. CORAL: expert-curated oncology reports to advance language model inference. NEJM AI. 2024;1:AIdbp2300110. 10.1056/aidbp2300110 PMC12007910 40255242

[26] Huang J , Yang DM, Rong R, et al A critical assessment of using ChatGPT for extracting structured data from clinical notes. NPJ Digit Med. 2024; 7:106. 10.1038/s41746-024-01079-8 38693429 PMC11063058

[27] Rae JW , Borgeaud S, Cai T, et al Scaling language models: methods, analysis & insights from training gopher. arXiv, arXiv:2112.11446, 2021, preprint: not peer reviewed.

[28] GrattafioriA DA , Jauhri A, et al The Llama 3 Herd of Models. arXiv, arXiv:2407.21783, 2024, preprint: not peer reviewed.

[29] Li C , Zhang M, He Y. The stability-efficiency dilemma: investigating sequence length warmup for training GPT models. Adv Neural Inf Process Syst. 2022;35:26736-26750.

