# Supplementary material for: Converting unstructured cardiac catheterization and echocardiography reports into structured data using transformer-based language models
Source: JAMIA Open. 2026 Mar 26;9(2):ooag036. doi: 10.1093/jamiaopen/ooag036 (PMC13020537; doi:10.1093/jamiaopen/ooag036)
Supplement: ooag036_Supplementary_Data [file ooag036_supplementary_data.zip › 14-Mar-2026_021351_JAMIO-2025-0492-R2-Supplementary-File.docx]

Table S1. Echocardiography Label Categories/Questions.

| 1. "LVEF": "What is the left ventricular ejection fraction?" |
| --- |
| 1. "RVSP": "What is the right ventricular systolic pressure?" |
| 1. "RA Pressure": "What is the right atrial pressure?" |
| 1. "LV Size": "What is the size of the left ventricle?" |
| 1. "RV Size": "What is the size of the right ventricle?" |
| 1. "RV Function": "What is the right ventricular systolic function?" |
| 1. "LVH": "What is the severity of left ventricular hypertrophy?" |
| 1. "LAE": "What is the severity of left atrial enlargement?" |
| 1. "RAE": "What is the severity of right atrial enlargement?" |
| 1. "WMA": "What are the wall motion abnormalities?" |
| 1. "Aortic Stenosis": "What is the severity of aortic stenosis?" |
| 1. "Aortic Regurgitation": "What is the severity of aortic regurgitation?" |
| 1. "Mitral Stenosis": "What is the severity of mitral stenosis?" |
| 1. "Mitral Regurgitation": "What is the severity of mitral regurgitation?" |
| 1. "Tricuspid Stenosis": "What is the severity of tricuspid stenosis?" |
| 1. "Tricuspid Regurgitation": "What is the severity of tricuspid regurgitation?" |
| 1. "Pulmonic Stenosis": "What is the severity of pulmonic stenosis?" |
| 1. "Pulmonic Regurgitation": "What is the severity of pulmonic regurgitation?" |
| 1. "Pulm HTN": "What is the severity of pulmonary hypertension?" |
| 1. "Pericardial Effusion": "What is the size of the pericardial effusion?" |
| 1. "Prosthetic Aortic Valve": "What prosthetic aortic valve is present?" |
| 1. "Prosthetic Mitral Valve": "What prosthetic mitral valve is present?" |
| 1. "Prosthetic Tricuspid Valve": "What prosthetic tricuspid valve is present?" |
| 1. "Prosthetic Pulmonic Valve": "What prosthetic pulmonic valve is present?" |
| 1. "Diastolic Dysfunction": "What is the grade of diastolic dysfunction?" |

Table S2. Cardiac Catheterization Label Categories/Questions.

| 1. "Left Main": "What is the most severe disease in the left main?" |
| --- |
| 1. "LAD": "What is the most severe disease in the left anterior descending artery?" |
| 1. "Diagonal 1": "What is the most severe disease in diagonal 1?" |
| 1. "Diagonal 2": "What is the most severe disease in diagonal 2?" |
| 1. "Diagonal 3": "What is the most severe disease in diagonal 3?" |
| 1. "Ramus": "What is the most severe disease in the ramus?" |
| 1. "LCX": "What is the most severe disease in the left circumflex coronary artery?" |
| 1. "OM1": "What is the most severe disease in the obtuse marginal 1?" |
| 1. "OM2": "What is the most severe disease in the obtuse marginal 2?" |
| 1. "OM3": "What is the most severe disease in the obtuse marginal 3?" |
| 1. "OM4": "What is the most severe disease in the obtuse marginal 4?" |
| 1. "OM5": "What is the most severe disease in the obtuse marginal 5?" |
| 1. "RCA": "What is the most severe disease in the right coronary artery?" |
| 1. "PDA": "What is the most severe disease in the posterior descending artery?" |
| 1. "PLV": "What is the most severe disease in the posterior left ventricular artery?" |
| 1. "LIMA-LAD": "What is the most severe disease in the left internal mammary artery-left anterior descending artery?" |
| 1. "LIMA-Diag": "What is the most severe disease in the left internal mammary artery-diagonal?" |
| 1. "LIMA-OM": "What is the most severe disease in the left internal mammary artery-obtuse marginal?" |
| 1. "LIMA-Ramus": "What is the most severe disease in the left internal mammary artery-ramus?" |
| 1. "SVGs": "What is the most severe disease in the saphenous vein grafts?" |
| 1. "SVG-OM1": "What is the most severe disease in the saphenous vein graft-obtuse marginal 1?" |
| 1. "SVG-OM2": "What is the most severe disease in the saphenous vein graft-obtuse marginal 2?" |
| 1. "SVG-OM3": "What is the most severe disease in the saphenous vein graft-obtuse marginal 3?" |
| 1. "SVG-OM4": "What is the most severe disease in the saphenous vein graft-obtuse marginal 4?" |
| 1. "SVG-LCX": "What is the most severe disease in the saphenous vein graft-left circumflex coronary artery?" |
| 1. "SVG-D1": "What is the most severe disease in the saphenous vein graft-diagonal 1?" |
| 1. "SVG-D2": "What is the most severe disease in the saphenous vein graft-diagonal 2?" |
| 1. "SVG-Ramus": "What is the most severe disease in the saphenous vein graft-ramus?" |
| 1. "SVG-RCA": "What is the most severe disease in the saphenous vein graft-right coronary artery?" |
| 1. "SVG-PDA": "What is the most severe disease in the saphenous vein graft-posterior descending artery?" |
| 1. "SVG-PLV": "What is the most severe disease in the saphenous vein graft-posterior left ventricular artery?" |
| 1. "SVG-LAD": "What is the most severe disease in the saphenous vein graft-left anterior descending artery?" |
| 1. "SVG-AM": "What is the most severe disease in the saphenous vein graft-acute marginal artery?” |
| 1. "SVG-LM": "What is the most severe disease in the saphenous vein graft-left main?" |
| 1. "RIMA-LAD": "What is the most severe disease in the right internal mammary artery-left anterior descending artery?" |
| 1. "RIMA-Diag": "What is the most severe disease in the right internal mammary artery-diagonal?" |
| 1. "RIMA-OM": "What is the most severe disease in the right internal mammary artery-obtuse marginal?" |
| 1. "RIMA-Ramus": "What is the most severe disease in the right internal mammary artery-ramus?" |
| 1. "RIMA-PLV": "What is the most severe disease in the right internal mammary artery-posterior left ventricular artery?" |
| 1. "RIMA": "What is the most severe disease in the right internal mammary artery?" |
| 1. "Radial-OM": "What is the most severe disease in the radial artery-obtuse marginal?" |
| 1. "Radial-LAD": "What is the most severe disease in the radial artery-left anterior descending artery?" |
| 1. "Radial-Diag": "What is the most severe disease in the radial artery-diagonal?" |
| 1. "Radial-Ramus": "What is the most severe disease in the radial artery-ramus?" |
| 1. "Radial-PDA": "What is the most severe disease in the radial artery-posterior descending artery?" |
| 1. "Radial-PLV": "What is the most severe disease in the radial artery-posterior left ventricular artery?" |
| 1. "Radial": "What is the most severe disease in the radial artery graft?" |

Table S3. The number of each labeled category with annotated values in the training and validation datasets of echocardiograph reports.

| Category | Training (n=2986) | Validation (n=300) |
| --- | --- | --- |
| Aortic regurgitation | 1084 | 101 |
| Aortic stenosis | 738 | 80 |
| Diastolic dysfunction | 978 | 94 |
| LAE | 1564 | 161 |
| LV size | 1972 | 198 |
| LVEF | 2802 | 281 |
| LVH | 1614 | 154 |
| Mitral regurgitation | 1452 | 136 |
| Mitral stenosis | 172 | 20 |
| Pericardial effusion | 1849 | 189 |
| Prosthetic aortic valve | 255 | 31 |
| Prosthetic mitral valve | 116 | 6 |
| Prosthetic tricuspid valve | 0 | 0 |
| Prosthetic pulmonic valve | 0 | 0 |
| Pulmonic HTN | 205 | 15 |
| Pulmonic regurgitation | 198 | 26 |
| Pulmonic stenosis | 3 | 2 |
| RA pressure | 528 | 56 |
| RAE | 1046 | 105 |
| RV function | 997 | 92 |
| RV size | 1006 | 94 |
| RVSP | 1459 | 144 |
| Tricuspid regurgitation | 1445 | 140 |
| Tricuspid stenosis | 11 | 0 |
| WMA | 1427 | 137 |

Table S4. The number of each labeled category with annotated values in the training and validation datasets of cardiac catheterization reports.

| Category | Training (n=1584) | Validation (n=300) |
| --- | --- | --- |
| Left Main | 1519 | 285 |
| LAD | 1518 | 289 |
| Diagonal 1 | 676 | 129 |
| Diagonal 2 | 268 | 62 |
| Diagonal 3 | 55 | 15 |
| Ramus | 270 | 43 |
| LCX | 1385 | 261 |
| OM1 | 696 | 126 |
| OM2 | 328 | 44 |
| OM3 | 96 | 22 |
| OM4 | 20 | 1 |
| OM5 | 6 | 1 |
| RCA | 1503 | 277 |
| PDA | 590 | 103 |
| PLV | 424 | 91 |
| LIMA-LAD | 579 | 3 |
| LIMA-Diag | 21 | 0 |
| LIMA-OM | 6 | 0 |
| LIMA-Ramus | 1 | 0 |
| SVGs | 49 | 0 |
| SVG-OM1 | 329 | 2 |
| SVG-OM2 | 72 | 0 |
| SVG-OM3 | 14 | 0 |
| SVG-OM4 | 0 | 0 |
| SVG-LCX | 9 | 0 |
| SVG-D1 | 154 | 1 |
| SVG-D2 | 25 | 0 |
| SVG-Ramus | 48 | 0 |
| SVG-RCA | 85 | 0 |
| SVG-PDA | 271 | 1 |
| SVG-PLV | 54 | 0 |
| SVG-LAD | 33 | 0 |
| SVG-AM | 3 | 0 |
| SVG-LM | 0 | 0 |
| RIMA-LAD | 7 | 0 |
| RIMA-Diag | 4 | 0 |
| RIMA-OM | 4 | 0 |
| RIMA-Ramus | 2 | 0 |
| RIMA-PLV | 5 | 0 |
| RIMA | 1 | 0 |
| Radial-OM | 7 | 0 |
| Radial-LAD | 0 | 0 |
| Radial-Diag | 1 | 0 |
| Radial-Ramus | 2 | 0 |
| Radial-PDA | 4 | 0 |
| Radial-PLV | 1 | 0 |
| Radial | 0 | 0 |

Table S5. The performance of the fine-tuned BioclinicalBERT and BART-Large-CNN models at the rand seeds of 42, 736, 299, 971, 243 against the manual annotated results in the echocardiography validation report dataset (n=300) at 0.1 and 0.5 cutoff thresholds of probability. The model was trained on 2986 reports with sequence length=512, batch size=4 and epochs=8.

| Extracted outcome^¥^ | BioclinicalBERT, Cutoff threshold of probability=0.1 | | | | | | | | | | | | | | | | | | | |
| --- | --- | --- | --- | --- | --- | --- | --- | --- | --- | --- | --- | --- | --- | --- | --- | --- | --- | --- | --- | --- |
|  | Accuracy (%) | | | | | Precision (%) | | | | | Recall (%) | | | | | F1score | | | | |
|  | Rand Seed=42 | Rand Seed=736 | Rand Seed=299 | Rand Seed=971 | Rand Seed=243 | Rand Seed= 42 | Rand Seed=736 | Rand Seed=299 | Rand Seed=971 | Rand Seed=243 | Rand Seed=42 | Rand Seed=736 | Rand Seed=299 | Rand Seed=971 | Rand Seed=243 | Rand Seed=42 | Rand Seed=736 | Rand Seed=299 | Rand Seed=971 | Rand Seed=243 |
| Aortic regurgitation | 97.12 | 96.12 | 96.15 | 96.15 | 98.06 | 97.12 | 98.02 | 96.15 | 97.09 | 98.06 | 100.00 | 98.02 | 99.01 | 99.01 | 100.00 | 0.99 | 0.98 | 0.98 | 0.98 | 0.99 |
| Aortic stenosis | 95.12 | 97.56 | 95.12 | 96.34 | 95.12 | 96.30 | 97.56 | 96.30 | 96.34 | 97.50 | 97.50 | 100.00 | 97.50 | 98.75 | 97.50 | 0.97 | 0.99 | 0.97 | 0.98 | 0.98 |
| Diastolic dysfunction | 95.79 | 95.79 | 94.74 | 94.79 | 95.79 | 97.85 | 97.85 | 96.77 | 96.81 | 97.85 | 96.81 | 96.81 | 95.74 | 96.81 | 96.81 | 0.97 | 0.97 | 0.96 | 0.97 | 0.97 |
| LAE | 97.55 | 96.95 | 96.93 | 97.55 | 96.93 | 98.76 | 98.15 | 98.75 | 98.76 | 98.14 | 98.76 | 98.76 | 98.14 | 98.76 | 98.14 | 0.99 | 0.98 | 0.98 | 0.99 | 0.98 |
| LV size | 95.00 | 96.00 | 95.50 | 95.50 | 95.50 | 97.44 | 97.46 | 97.45 | 97.45 | 97.45 | 95.96 | 96.97 | 96.46 | 96.46 | 96.46 | 0.97 | 0.97 | 0.97 | 0.97 | 0.97 |
| LVEF | 98.94 | 98.94 | 98.58 | 99.29 | 98.94 | 99.29 | 99.64 | 99.29 | 99.64 | 99.29 | 99.29 | 99.29 | 98.93 | 99.64 | 99.29 | 0.99 | 0.99 | 0.99 | 1.00 | 0.99 |
| LVH | 96.79 | 97.44 | 98.08 | 96.82 | 97.44 | 98.05 | 98.06 | 98.08 | 97.44 | 98.06 | 98.05 | 98.70 | 99.35 | 98.70 | 98.70 | 0.98 | 0.98 | 0.99 | 0.98 | 0.98 |
| Mitral regurgitation | 97.81 | 99.27 | 97.81 | 99.27 | 98.54 | 98.53 | 99.27 | 98.53 | 99.27 | 99.26 | 98.53 | 100.00 | 98.53 | 100.00 | 99.26 | 0.99 | 1.00 | 0.99 | 1.00 | 0.99 |
| Mitral stenosis | 80.95 | 90.91 | 90.48 | 79.17 | 86.36 | 89.47 | 90.91 | 90.48 | 79.17 | 86.36 | 85.00 | 100.00 | 95.00 | 95.00 | 95.00 | 0.87 | 0.95 | 0.93 | 0.86 | 0.90 |
| Pericardial effusion | 96.88 | 96.88 | 96.89 | 96.89 | 96.88 | 97.89 | 97.89 | 97.40 | 97.40 | 97.89 | 98.41 | 98.41 | 98.94 | 98.94 | 98.41 | 0.98 | 0.98 | 0.98 | 0.98 | 0.98 |
| Prosthetic aortic valve | 93.55 | 93.55 | 90.63 | 93.55 | 90.32 | 100.00 | 100.00 | 96.67 | 100.00 | 100.00 | 93.55 | 93.55 | 93.55 | 93.55 | 90.32 | 0.97 | 0.97 | 0.95 | 0.97 | 0.95 |
| Prosthetic mitral valve | 66.67 | 66.67 | 66.67 | 57.14 | 66.67 | 100.00 | 100.00 | 100.00 | 80.00 | 100.00 | 66.67 | 66.67 | 66.67 | 66.67 | 66.67 | 0.80 | 0.80 | 0.80 | 0.73 | 0.80 |
| Pulmonic HTN | 100.00 | 93.33 | 100.00 | 93.33 | 100.00 | 100.00 | 100.00 | 100.00 | 100.00 | 100.00 | 100.00 | 93.33 | 100.00 | 93.33 | 100.00 | 1.00 | 0.97 | 1.00 | 0.97 | 1.00 |
| Pulmonic regurgitation | 92.86 | 96.30 | 100.00 | 96.30 | 96.30 | 92.86 | 96.30 | 100.00 | 96.30 | 96.30 | 100.00 | 100.00 | 100.00 | 100.00 | 100.00 | 0.96 | 0.98 | 1.00 | 0.98 | 0.98 |
| Pulmonic stenosis | 50.00 | 50.00 | 50.00 | 50.00 | 50.00 | 100.00 | 100.00 | 100.00 | 100.00 | 100.00 | 50.00 | 50.00 | 50.00 | 50.00 | 50.00 | 0.67 | 0.67 | 0.67 | 0.67 | 0.67 |
| RA pressure | 86.21 | 82.76 | 84.48 | 81.03 | 84.48 | 96.15 | 96.00 | 96.08 | 94.00 | 96.08 | 89.29 | 85.71 | 87.50 | 83.93 | 87.50 | 0.93 | 0.91 | 0.92 | 0.89 | 0.92 |
| RAE | 96.26 | 96.26 | 95.33 | 96.26 | 96.26 | 98.10 | 98.10 | 98.08 | 98.10 | 98.10 | 98.10 | 98.10 | 97.14 | 98.10 | 98.10 | 0.98 | 0.98 | 0.98 | 0.98 | 0.98 |
| RV function | 93.55 | 90.63 | 92.55 | 91.40 | 92.47 | 97.75 | 94.57 | 96.67 | 97.70 | 97.73 | 94.57 | 94.57 | 94.57 | 92.39 | 93.48 | 0.96 | 0.95 | 0.96 | 0.95 | 0.96 |
| RV size | 89.22 | 89.22 | 90.20 | 87.25 | 92.00 | 91.92 | 91.00 | 92.00 | 90.82 | 93.88 | 96.81 | 96.81 | 97.87 | 94.68 | 97.87 | 0.94 | 0.94 | 0.95 | 0.93 | 0.96 |
| RVSP | 97.26 | 95.95 | 97.95 | 96.60 | 96.58 | 97.26 | 95.95 | 97.95 | 96.60 | 97.92 | 98.61 | 98.61 | 99.31 | 98.61 | 97.92 | 0.98 | 0.97 | 0.99 | 0.98 | 0.98 |
| Tricuspid regurgitation | 99.29 | 98.57 | 97.86 | 97.87 | 99.29 | 100.00 | 100.00 | 99.28 | 99.28 | 100.00 | 99.29 | 98.57 | 97.86 | 98.57 | 99.29 | 1.00 | 0.99 | 0.99 | 0.99 | 1.00 |
| WMA | 92.86 | 91.49 | 90.78 | 92.20 | 92.14 | 97.74 | 96.99 | 96.97 | 97.01 | 97.73 | 94.89 | 94.16 | 93.43 | 94.89 | 94.16 | 0.96 | 0.96 | 0.95 | 0.96 | 0.96 |
| *All* | 95.78 | 95.66 | 95.61 | 95.37 | 95.91 | 97.74 | 97.61 | 97.56 | 97.35 | 97.95 | 97.39 | 97.52 | 97.30 | 97.35 | 97.39 | 0.98 | 0.98 | 0.97 | 0.97 | 0.98 |
|  | BioclinicalBERT, Cutoff threshold of probability=0.5 | | | | | | | | | | | | | | | | | | | |
|  | Accuracy (%) | | | | | Precision (%) | | | | | Recall (%) | | | | | F1score | | | | |
|  | Rand Seed=42 | Rand Seed=736 | Rand Seed=299 | Rand Seed=971 | Rand Seed=243 | Rand Seed=42 | Rand Seed=736 | Rand Seed=299 | Rand Seed=971 | Rand Seed=243 | Rand Seed=42 | Rand Seed=736 | Rand Seed=299 | Rand Seed=971 | Rand Seed=243 | Rand Seed=42 | Rand Seed=736 | Rand Seed=299 | Rand Seed=971 | Rand Seed=243 |
| Aortic regurgitation | 95.19 | 96.12 | 96.15 | 96.15 | 96.12 | 97.06 | 98.02 | 97.09 | 97.09 | 98.02 | 98.02 | 98.02 | 99.01 | 99.01 | 98.02 | 0.98 | 0.98 | 0.98 | 0.98 | 0.98 |
| Aortic stenosis | 95.12 | 97.56 | 95.12 | 96.34 | 95.12 | 97.50 | 97.56 | 96.30 | 97.53 | 97.50 | 97.50 | 100.00 | 97.50 | 98.75 | 97.50 | 0.98 | 0.99 | 0.97 | 0.98 | 0.98 |
| Diastolic dysfunction | 95.79 | 95.79 | 94.74 | 94.79 | 95.79 | 97.85 | 97.85 | 96.77 | 96.81 | 97.85 | 96.81 | 96.81 | 95.74 | 96.81 | 96.81 | 0.97 | 0.97 | 0.96 | 0.97 | 0.97 |
| LAE | 97.55 | 96.34 | 96.93 | 97.55 | 96.93 | 98.76 | 98.14 | 98.75 | 98.76 | 98.14 | 98.76 | 98.14 | 98.14 | 98.76 | 98.14 | 0.99 | 0.98 | 0.98 | 0.99 | 0.98 |
| LV size | 95.00 | 96.00 | 95.50 | 95.50 | 95.50 | 97.44 | 97.46 | 97.45 | 97.45 | 97.45 | 95.96 | 96.97 | 96.46 | 96.46 | 96.46 | 0.97 | 0.97 | 0.97 | 0.97 | 0.97 |
| LVEF | 98.58 | 98.58 | 98.23 | 98.94 | 98.58 | 99.29 | 99.64 | 99.28 | 99.64 | 99.29 | 98.93 | 98.93 | 98.58 | 99.29 | 98.93 | 0.99 | 0.99 | 0.99 | 0.99 | 0.99 |
| LVH | 96.79 | 96.79 | 96.15 | 96.18 | 96.79 | 98.05 | 98.05 | 98.04 | 97.42 | 98.05 | 98.05 | 98.05 | 97.40 | 98.05 | 98.05 | 0.98 | 0.98 | 0.98 | 0.98 | 0.98 |
| Mitral regurgitation | 96.35 | 99.27 | 96.35 | 99.27 | 98.54 | 99.25 | 99.27 | 98.51 | 99.27 | 99.26 | 97.06 | 100.00 | 97.06 | 100.00 | 99.26 | 0.98 | 1.00 | 0.98 | 1.00 | 0.99 |
| Mitral stenosis | 80.95 | 90.91 | 90.48 | 82.61 | 86.36 | 89.47 | 90.91 | 90.48 | 82.61 | 86.36 | 85.00 | 100.0 | 95.00 | 95.00 | 95.00 | 0.87 | 0.95 | 0.93 | 0.88 | 0.90 |
| Pericardial effusion | 96.88 | 96.35 | 95.85 | 97.40 | 96.88 | 97.89 | 97.88 | 97.88 | 97.91 | 97.89 | 98.41 | 97.88 | 97.88 | 98.94 | 98.41 | 0.98 | 0.98 | 0.98 | 0.98 | 0.98 |
| Prosthetic aortic valve | 93.55 | 93.55 | 93.55 | 90.32 | 90.32 | 100.00 | 100.00 | 100.00 | 100.00 | 100.00 | 93.55 | 93.55 | 93.55 | 90.32 | 90.32 | 0.97 | 0.97 | 0.97 | 0.95 | 0.95 |
| Prosthetic mitral valve | 66.67 | 66.67 | 66.67 | 57.14 | 66.67 | 100.00 | 100.00 | 100.00 | 80.00 | 100.00 | 66.67 | 66.67 | 66.67 | 66.67 | 66.67 | 0.80 | 0.80 | 0.80 | 0.73 | 0.80 |
| Pulmonic HTN | 100.00 | 93.33 | 100.00 | 93.33 | 100.00 | 100.00 | 100.00 | 100.00 | 100.00 | 100.00 | 100.00 | 93.33 | 100.00 | 93.33 | 100.00 | 1.00 | 0.97 | 1.00 | 0.97 | 1.00 |
| Pulmonic regurgitation | 89.29 | 96.30 | 100.00 | 96.30 | 96.30 | 92.59 | 96.30 | 100.00 | 96.30 | 96.30 | 96.15 | 100.00 | 100.00 | 100.00 | 100.00 | 0.94 | 0.98 | 1.00 | 0.98 | 0.98 |
| Pulmonic stenosis | 50.00 | 50.00 | 50.00 | 50.00 | 50.00 | 100.00 | 100.00 | 100.00 | 100.00 | 100.00 | 50.00 | 50.00 | 50.00 | 50.00 | 50.00 | 0.67 | 0.67 | 0.67 | 0.67 | 0.67 |
| RA pressure | 86.21 | 82.76 | 82.76 | 81.03 | 84.48 | 96.15 | 96.00 | 96.00 | 95.92 | 96.08 | 89.29 | 85.71 | 85.71 | 83.93 | 87.50 | 0.93 | 0.91 | 0.91 | 0.90 | 0.92 |
| RAE | 96.26 | 96.26 | 95.33 | 96.26 | 96.26 | 98.10 | 98.10 | 98.08 | 98.10 | 98.10 | 98.10 | 98.10 | 97.14 | 98.10 | 98.10 | 0.98 | 0.98 | 0.98 | 0.98 | 0.98 |
| RV function | 92.47 | 91.58 | 92.55 | 91.40 | 91.40 | 97.73 | 95.60 | 96.67 | 97.70 | 97.70 | 93.48 | 94.57 | 94.57 | 92.39 | 92.39 | 0.96 | 0.95 | 0.96 | 0.95 | 0.95 |
| RV size | 90.10 | 89.22 | 90.20 | 87.25 | 92.00 | 92.86 | 91.00 | 92.00 | 91.75 | 93.88 | 96.81 | 96.81 | 97.87 | 94.68 | 97.87 | 0.95 | 0.94 | 0.95 | 0.93 | 0.96 |
| RVSP | 97.26 | 95.95 | 97.95 | 96.60 | 96.58 | 97.26 | 95.95 | 97.95 | 96.60 | 97.92 | 98.61 | 98.61 | 99.31 | 98.61 | 97.92 | 0.98 | 0.97 | 0.99 | 0.98 | 0.98 |
| Tricuspid regurgitation | 98.57 | 98.57 | 97.14 | 97.87 | 99.29 | 100.00 | 100.00 | 99.27 | 99.28 | 100.00 | 98.57 | 98.57 | 97.14 | 98.57 | 99.29 | 0.99 | 0.99 | 0.98 | 0.99 | 1.00 |
| WMA | 92.86 | 90.07 | 89.36 | 91.49 | 90.00 | 97.74 | 96.95 | 96.92 | 96.99 | 97.67 | 94.89 | 92.70 | 91.97 | 94.16 | 91.97 | 0.96 | 0.95 | 0.94 | 0.96 | 0.95 |
| *All* | 95.48 | 95.44 | 95.13 | 95.28 | 95.56 | 97.86 | 97.65 | 97.68 | 97.56 | 97.95 | 97.04 | 97.26 | 96.77 | 97.17 | 97.04 | 0.97 | 0.97 | 0.97 | 0.97 | 0.97 |
|  | BART-Large-CNN, Cutoff threshold of probability=0.1 | | | | | | | | | | | | | | | | | | | |
|  | Accuracy (%) | | | | | Precision (%) | | | | | Recall (%) | | | | | F1score | | | | |
|  | Rand Seed=42 | Rand Seed=736 | Rand Seed=299 | Rand Seed=971 | Rand Seed=243 | Rand Seed=42 | Rand Seed=736 | Rand Seed=299 | Rand Seed=971 | Rand Seed=243 | Rand Seed=42 | Rand Seed=736 | Rand Seed=299 | Rand Seed=971 | Rand Seed=243 | Rand Seed=42 | Rand Seed=736 | Rand Seed=299 | Rand Seed=971 | Rand Seed=243 |
| Aortic regurgitation | 97.12 | 97.12 | 97.12 | 96.19 | 97.12 | 97.12 | 97.12 | 97.12 | 96.19 | 97.12 | 100.00 | 100.00 | 100.00 | 100.00 | 100.00 | 0.99 | 0.99 | 0.99 | 0.98 | 0.99 |
| Aortic stenosis | 97.56 | 96.34 | 96.34 | 97.56 | 96.34 | 97.56 | 97.53 | 97.53 | 97.56 | 97.53 | 100.00 | 98.75 | 98.75 | 100.00 | 98.75 | 0.99 | 0.98 | 0.98 | 0.99 | 0.98 |
| Diastolic dysfunction | 95.79 | 95.83 | 95.79 | 95.83 | 94.74 | 97.85 | 96.84 | 97.85 | 96.84 | 97.83 | 96.81 | 97.87 | 96.81 | 97.87 | 95.74 | 0.97 | 0.97 | 0.97 | 0.97 | 0.97 |
| LAE | 96.95 | 97.55 | 97.56 | 96.95 | 96.95 | 98.15 | 98.76 | 98.16 | 98.15 | 97.55 | 98.76 | 98.76 | 99.38 | 98.76 | 98.76 | 0.98 | 0.99 | 0.99 | 0.98 | 0.98 |
| LV size | 96.48 | 95.00 | 95.00 | 95.00 | 95.48 | 98.97 | 96.94 | 96.94 | 96.94 | 97.94 | 96.97 | 95.96 | 95.96 | 95.96 | 95.96 | 0.98 | 0.96 | 0.96 | 0.96 | 0.97 |
| LVEF | 98.58 | 98.94 | 98.94 | 99.29 | 98.94 | 98.93 | 99.29 | 99.29 | 99.64 | 99.64 | 98.93 | 99.29 | 99.29 | 99.64 | 99.29 | 0.99 | 0.99 | 0.99 | 1.00 | 0.99 |
| LVH | 98.08 | 96.79 | 97.44 | 97.44 | 97.44 | 98.71 | 97.42 | 98.06 | 98.06 | 98.06 | 99.35 | 98.05 | 98.70 | 98.70 | 98.7 | 0.99 | 0.98 | 0.98 | 0.98 | 0.98 |
| Mitral regurgitation | 97.81 | 98.54 | 97.81 | 97.08 | 97.81 | 99.26 | 98.54 | 98.53 | 98.52 | 99.26 | 98.53 | 99.26 | 98.53 | 97.79 | 98.53 | 0.99 | 0.99 | 0.99 | 0.98 | 0.99 |
| Mitral stenosis | 95.24 | 90.48 | 86.36 | 90.91 | 86.36 | 95.24 | 90.48 | 86.36 | 90.91 | 86.36 | 100.00 | 95.00 | 95.00 | 100.00 | 95.00 | 0.98 | 0.93 | 0.90 | 0.95 | 0.90 |
| Pericardial effusion | 96.34 | 97.38 | 96.86 | 97.91 | 97.91 | 98.40 | 98.94 | 98.40 | 98.94 | 98.94 | 97.35 | 98.41 | 97.88 | 98.94 | 98.94 | 0.98 | 0.99 | 0.98 | 0.99 | 0.99 |
| Prosthetic aortic valve | 84.38 | 93.55 | 93.55 | 90.63 | 90.63 | 96.43 | 100.00 | 100.00 | 96.67 | 96.67 | 87.10 | 93.55 | 93.55 | 93.55 | 93.55 | 0.92 | 0.97 | 0.97 | 0.95 | 0.95 |
| Prosthetic mitral valve | 66.67 | 66.67 | 66.67 | 66.67 | 66.67 | 100.00 | 100.00 | 100.00 | 100.00 | 100.00 | 66.67 | 66.67 | 66.67 | 66.67 | 66.67 | 0.80 | 0.80 | 0.80 | 0.80 | 0.80 |
| Pulmonic HTN | 93.33 | 100.00 | 100.00 | 100.00 | 93.33 | 100.00 | 100.00 | 100.00 | 100.00 | 100.00 | 93.33 | 100.00 | 100.00 | 100.00 | 93.33 | 0.97 | 1.00 | 1.00 | 1.00 | 0.97 |
| Pulmonic regurgitation | 96.30 | 100.00 | 96.30 | 100.00 | 100.00 | 96.30 | 100.00 | 96.30 | 100.00 | 100.00 | 100.00 | 100.00 | 100.00 | 100.00 | 100.00 | 0.98 | 1.00 | 0.98 | 1.00 | 1.00 |
| Pulmonic stenosis | 50.00 | 50.00 | 50.00 | 50.00 | 50.00 | 100.00 | 100.00 | 100.00 | 100.00 | 100.00 | 50.00 | 50.00 | 50.00 | 50.00 | 50.00 | 0.67 | 0.67 | 0.67 | 0.67 | 0.67 |
| RA pressure | 82.76 | 83.33 | 84.75 | 86.67 | 86.21 | 94.12 | 92.59 | 94.34 | 92.86 | 96.15 | 85.71 | 89.29 | 89.29 | 92.86 | 89.29 | 0.9 | 0.91 | 0.92 | 0.93 | 0.93 |
| RAE | 97.17 | 96.26 | 96.26 | 96.26 | 96.26 | 99.04 | 98.10 | 98.10 | 98.10 | 98.10 | 98.10 | 98.10 | 98.10 | 98.10 | 98.10 | 0.99 | 0.98 | 0.98 | 0.98 | 0.98 |
| RV function | 93.55 | 89.47 | 92.47 | 91.58 | 93.55 | 97.75 | 95.51 | 97.73 | 95.60 | 97.75 | 94.57 | 92.39 | 93.48 | 94.57 | 94.57 | 0.96 | 0.94 | 0.96 | 0.95 | 0.96 |
| RV size | 87.25 | 86.27 | 89.11 | 90.20 | 89.22 | 91.75 | 90.72 | 91.84 | 91.09 | 91.92 | 94.68 | 93.62 | 95.74 | 97.87 | 96.81 | 0.93 | 0.92 | 0.94 | 0.94 | 0.94 |
| RVSP | 96.60 | 97.26 | 97.26 | 95.97 | 96.58 | 97.26 | 97.93 | 97.93 | 95.97 | 97.92 | 98.61 | 98.61 | 98.61 | 99.31 | 97.92 | 0.98 | 0.98 | 0.98 | 0.98 | 0.98 |
| Tricuspid regurgitation | 97.87 | 98.58 | 98.58 | 98.58 | 99.29 | 98.57 | 99.29 | 99.29 | 99.29 | 99.29 | 98.57 | 99.29 | 99.29 | 99.29 | 100.00 | 0.99 | 0.99 | 0.99 | 0.99 | 1.00 |
| WMA | 90.78 | 90.14 | 92.14 | 89.58 | 90.85 | 96.97 | 96.24 | 97.73 | 94.85 | 94.85 | 93.43 | 93.43 | 94.16 | 94.16 | 94.16 | 0.95 | 0.95 | 0.96 | 0.95 | 0.95 |
| *All* | 95.57 | 95.49 | 95.78 | 95.68 | 95.79 | 97.86 | 97.52 | 97.70 | 97.28 | 97.74 | 97.21 | 97.30 | 97.44 | 97.88 | 97.48 | 0.98 | 0.97 | 0.98 | 0.98 | 0.98 |
|  | BART-Large-CNN, Cutoff threshold of probability=0.5 | | | | | | | | | | | | | | | | | | | |
|  | Accuracy (%) | | | | | Precision (%) | | | | | Recall (%) | | | | | F1score | | | | |
|  | Rand Seed=42 | Rand Seed=736 | Rand Seed=299 | Rand Seed=971 | Rand Seed=243 | Rand Seed=42 | Rand Seed=736 | Rand Seed=299 | Rand Seed=971 | Rand Seed=243 | Rand Seed=42 | Rand Seed=736 | Rand Seed=299 | Rand Seed=971 | Rand Seed=243 | Rand Seed=42 | Rand Seed=736 | Rand Seed=299 | Rand Seed=971 | Rand Seed=243 |
| Aortic regurgitation | 97.12 | 97.12 | 97.12 | 96.19 | 97.12 | 97.12 | 97.12 | 97.12 | 96.19 | 97.12 | 100.00 | 100.00 | 100.00 | 100.00 | 100.00 | 0.99 | 0.99 | 0.99 | 0.98 | 0.99 |
| Aortic stenosis | 96.34 | 96.34 | 96.34 | 95.12 | 96.34 | 97.53 | 97.53 | 97.53 | 96.30 | 97.53 | 98.75 | 98.75 | 98.75 | 97.5 | 98.75 | 0.98 | 0.98 | 0.98 | 0.97 | 0.98 |
| Diastolic dysfunction | 95.79 | 95.83 | 95.79 | 95.83 | 94.74 | 97.85 | 96.84 | 97.85 | 96.84 | 97.83 | 96.81 | 97.87 | 96.81 | 97.87 | 95.74 | 0.97 | 0.97 | 0.97 | 0.97 | 0.97 |
| LAE | 96.95 | 97.55 | 97.56 | 96.95 | 96.34 | 98.15 | 98.76 | 98.16 | 98.15 | 97.53 | 98.76 | 98.76 | 99.38 | 98.76 | 98.14 | 0.98 | 0.99 | 0.99 | 0.98 | 0.98 |
| LV size | 96.48 | 95.00 | 95.00 | 95.00 | 95.48 | 98.97 | 97.44 | 97.44 | 96.94 | 97.94 | 96.97 | 95.96 | 95.96 | 95.96 | 95.96 | 0.98 | 0.97 | 0.97 | 0.96 | 0.97 |
| LVEF | 98.58 | 98.58 | 98.94 | 99.29 | 98.58 | 98.93 | 99.29 | 99.29 | 99.64 | 99.64 | 98.93 | 98.93 | 99.29 | 99.64 | 98.93 | 0.99 | 0.99 | 0.99 | 1.00 | 0.99 |
| LVH | 96.15 | 96.15 | 97.44 | 96.15 | 97.44 | 98.04 | 97.40 | 98.06 | 98.04 | 98.06 | 97.40 | 97.40 | 98.70 | 97.40 | 98.7 | 0.98 | 0.97 | 0.98 | 0.98 | 0.98 |
| Mitral regurgitation | 97.81 | 98.54 | 97.81 | 97.08 | 97.81 | 99.26 | 98.54 | 98.53 | 98.52 | 99.26 | 98.53 | 99.26 | 98.53 | 97.79 | 98.53 | 0.99 | 0.99 | 0.99 | 0.98 | 0.99 |
| Mitral stenosis | 95.24 | 90.48 | 86.36 | 90.91 | 90.48 | 95.24 | 95.00 | 86.36 | 90.91 | 90.48 | 100.00 | 95.00 | 95.00 | 100.00 | 95.00 | 0.98 | 0.95 | 0.90 | 0.95 | 0.93 |
| Pericardial effusion | 96.84 | 97.38 | 96.86 | 98.42 | 97.91 | 98.92 | 98.94 | 98.40 | 99.47 | 98.94 | 97.35 | 98.41 | 97.88 | 98.94 | 98.94 | 0.98 | 0.99 | 0.98 | 0.99 | 0.99 |
| Prosthetic aortic valve | 84.38 | 93.55 | 93.55 | 90.63 | 90.63 | 96.43 | 100.00 | 100.00 | 96.67 | 96.67 | 87.10 | 93.55 | 93.55 | 93.55 | 93.55 | 0.92 | 0.97 | 0.97 | 0.95 | 0.95 |
| Prosthetic mitral valve | 66.67 | 66.67 | 66.67 | 66.67 | 66.67 | 100.00 | 100.00 | 100.00 | 100.00 | 100.00 | 66.67 | 66.67 | 66.67 | 66.67 | 66.67 | 0.80 | 0.80 | 0.80 | 0.80 | 0.80 |
| Pulmonic HTN | 93.33 | 100.00 | 100.00 | 100.00 | 93.33 | 100.00 | 100.00 | 100.00 | 100.00 | 100.00 | 93.33 | 100.00 | 100.00 | 100.00 | 93.33 | 0.97 | 1.00 | 1.00 | 1.00 | 0.97 |
| Pulmonic regurgitation | 96.30 | 100.00 | 96.30 | 100.00 | 100.00 | 96.30 | 100.00 | 96.30 | 100.00 | 100.00 | 100.00 | 100.00 | 100.00 | 100.00 | 100.00 | 0.98 | 1.00 | 0.98 | 1.00 | 1.00 |
| Pulmonic stenosis | 0.00 | 50.00 | 50.00 | 50.00 | 50.00 | 0.00 | 100.00 | 100.00 | 100.00 | 100.00 | 0.00 | 50.00 | 50.00 | 50.00 | 50.00 | 0.00 | 0.67 | 0.67 | 0.67 | 0.67 |
| RA pressure | 82.76 | 84.75 | 84.75 | 86.67 | 86.21 | 96.00 | 94.34 | 94.34 | 92.86 | 96.15 | 85.71 | 89.29 | 89.29 | 92.86 | 89.29 | 0.91 | 0.92 | 0.92 | 0.93 | 0.93 |
| RAE | 97.17 | 96.26 | 96.26 | 96.26 | 95.33 | 99.04 | 98.10 | 98.10 | 98.10 | 98.08 | 98.10 | 98.10 | 98.10 | 98.10 | 97.14 | 0.99 | 0.98 | 0.98 | 0.98 | 0.98 |
| RV function | 92.47 | 89.47 | 92.47 | 91.58 | 93.55 | 97.73 | 95.51 | 97.73 | 95.60 | 97.75 | 93.48 | 92.39 | 93.48 | 94.57 | 94.57 | 0.96 | 0.94 | 0.96 | 0.95 | 0.96 |
| RV size | 86.27 | 85.29 | 89.11 | 88.24 | 88.24 | 91.67 | 90.63 | 91.84 | 90.91 | 91.84 | 93.62 | 92.55 | 95.74 | 95.74 | 95.74 | 0.93 | 0.92 | 0.94 | 0.93 | 0.94 |
| RVSP | 96.60 | 97.26 | 97.26 | 95.97 | 95.89 | 97.26 | 97.93 | 97.93 | 95.97 | 97.90 | 98.61 | 98.61 | 98.61 | 99.31 | 97.22 | 0.98 | 0.98 | 0.98 | 0.98 | 0.98 |
| Tricuspid regurgitation | 97.87 | 98.58 | 97.87 | 98.58 | 99.29 | 99.28 | 99.29 | 99.28 | 99.29 | 100.00 | 98.57 | 99.29 | 98.57 | 99.29 | 99.29 | 0.99 | 0.99 | 0.99 | 0.99 | 1.00 |
| WMA | 90.78 | 90.78 | 91.43 | 89.51 | 90.85 | 96.97 | 96.97 | 97.71 | 95.52 | 94.85 | 93.43 | 93.43 | 93.43 | 93.43 | 94.16 | 0.95 | 0.95 | 0.96 | 0.94 | 0.95 |
| *All* | 95.30 | 95.44 | 95.70 | 95.46 | 95.61 | 97.94 | 97.69 | 97.74 | 97.31 | 97.82 | 96.91 | 97.17 | 97.35 | 97.57 | 97.21 | 0.97 | 0.97 | 0.98 | 0.97 | 0.98 |

^¥^ The performance of Prosthetic tricuspid valve, Prosthetic pulmonic valve and Tricuspid stenosis was not evaluated because of no confirmed cases.

Table S6. The mean and standard deviation of the performance of the fine-tuned BioclinicalBERT and BART-Large-CNN models at five different rand seeds of 42, 736, 299, 971, 243 against the manual annotated results in the echocardiography validation report dataset (n=300) at 0.1 and 0.5 cutoff thresholds of probability. The model was trained on 2986 reports with sequence length=512, batch size=4 and epochs=8.

| Extracted outcome^¥^ | Cutoff threshold of probability=0.1 | | | | | | | | Cutoff threshold of probability=0.5 | | | | | | | |
| --- | --- | --- | --- | --- | --- | --- | --- | --- | --- | --- | --- | --- | --- | --- | --- | --- |
|  | BioclinicalBERT | | | | BART-Large-CNN | | | | BioclinicalBERT | | | | BART-Large-CNN | | | |
|  | Accuracy (%) | Precision (%) | Recall (%) | F1score | Accuracy (%) | Precision (%) | Recall (%) | F1score | Accuracy (%) | Precision (%) | Recall (%) | F1score | Accuracy (%) | Precision (%) | Recall (%) | F1score |
| Aortic regurgitation | 96.7(0.9) | 97.3(0.8) | 99.2(0.8) | 0.98(0.01) | 96.9(0.4) | 96.9(0.4) | 100.0(0.0) | 0.98(0.00) | 96.0(0.4) | 97.5(0.5) | 98.4(0.5) | 0.98(0.00) | 96.9(0.4) | 96.9(0.4) | 100.0(0.0) | 0.98(0.00) |
| Aortic stenosis | 95.9(1.0) | 96.8(0.7) | 98.3(1.1) | 0.98(0.01) | 96.8(0.7) | 97.5(0.0) | 99.3(0.7) | 0.98(0.00) | 95.9(1.1) | 97.3(0.6) | 98.3(1.1) | 0.98(0.01) | 96.1(0.6) | 97.3(0.6) | 98.5(0.6) | 0.98(0.01) |
| Diastolic dysfunction | 95.4(0.6) | 97.4(0.6) | 96.6(0.5) | 0.97(0.00) | 95.6(0.5) | 97.4(0.6) | 97.0(0.9) | 0.97(0.00) | 95.4(0.6) | 97.4(0.6) | 96.6(0.5) | 0.97(0.00) | 95.6(0.5) | 97.4(0.6) | 97.0(0.9) | 0.97(0.00) |
| LAE | 97.2(0.3) | 98.5(0.3) | 98.5(0.3) | 0.99(0.00) | 97.2(0.3) | 98.2(0.4) | 98.9(0.3) | 0.99(0.00) | 97.1(0.5) | 98.5(0.3) | 98.4(0.3) | 0.98(0.00) | 97.1(0.5) | 98.2(0.4) | 98.8(0.4) | 0.98(0.00) |
| LV size | 95.5(0.4) | 97.5(0.0) | 96.5(0.4) | 0.97(0.00) | 95.4(0.6) | 97.5(0.9) | 96.2(0.5) | 0.97(0.01) | 95.5(0.4) | 97.5(0.0) | 96.5(0.4) | 0.97(0.00) | 95.4(0.6) | 97.7(0.8) | 96.2(0.5) | 0.97(0.01) |
| LVEF | 98.9(0.3) | 99.4(0.2) | 99.3(0.3) | 0.99(0.00) | 98.9(0.3) | 99.4(0.3) | 99.3(0.3) | 0.99(0.00) | 98.6(0.3) | 99.4(0.2) | 98.9(0.3) | 0.99(0.00) | 98.8(0.3) | 99.4(0.3) | 99.2(0.3) | 0.99(0.00) |
| LVH | 97.3(0.5) | 97.9(0.3) | 98.7(0.5) | 0.98(0.00) | 97.4(0.5) | 98.1(0.5) | 98.7(0.5) | 0.98(0.00) | 96.5(0.3) | 97.9(0.3) | 97.9(0.3) | 0.98(0.00) | 96.7(0.7) | 97.9(0.3) | 97.9(0.7) | 0.98(0.00) |
| Mitral regurgitation | 98.5(0.7) | 99.0(0.4) | 99.3(0.7) | 0.99(0.01) | 97.8(0.5) | 98.8(0.4) | 98.5(0.5) | 0.99(0.00) | 98.0(1.5) | 99.1(0.3) | 98.7(1.5) | 0.99(0.01) | 97.8(0.5) | 98.8(0.4) | 98.5(0.5) | 0.99(0.00) |
| Mitral stenosis | 85.6(5.4) | 87.3(4.9) | 94.0(5.5) | 0.90(0.04) | 89.9(3.7) | 89.9(3.7) | 97.0(2.7) | 0.93(0.03) | 86.3(4.5) | 88.0(3.5) | 94.0(5.5) | 0.91(0.03) | 90.7(3.1) | 91.6(3.7) | 97.0(2.7) | 0.94(0.03) |
| Pericardial effusion | 96.9(0.0) | 97.7(0.3) | 98.6(0.3) | 0.98(0.00) | 97.3(0.7) | 98.7(0.3) | 98.3(0.7) | 0.99(0.00) | 96.7(0.6) | 97.9(0.0) | 98.3(0.4) | 0.98(0.00) | 97.5(0.7) | 98.9(0.4) | 98.3(0.7) | 0.99(0.00) |
| Prosthetic aortic valve | 92.3(1.7) | 99.3(1.5) | 92.9(1.4) | 0.96(0.01) | 90.5(3.8) | 98.0(1.9) | 92.3(2.9) | 0.95(0.02) | 92.3(1.8) | 100.0(0.0) | 92.3(1.8) | 0.96(0.01) | 90.5(3.8) | 98.0(1.9) | 92.3(2.9) | 0.95(0.02) |
| Prosthetic mitral valve | 64.8(4.3) | 96(8.9) | 66.7(0.0) | 0.79(0.03) | 66.7(0.0) | 100.0(0.0) | 66.7(0.0) | 0.80(0.00) | 64.8(4.3) | 96.0(8.9) | 66.7(0.0) | 0.79(0.03) | 66.7(0.0) | 100.0(0.0) | 66.7(0.0) | 0.80(0.00) |
| Pulmonic HTN | 97.3(3.7) | 100.0(0.0) | 97.3(3.7) | 0.99(0.02) | 97.3(3.7) | 100.0(0.0) | 97.3(3.7) | 0.99(0.02) | 97.3(3.7) | 100.0(0.0) | 97.3(3.7) | 0.99(0.02) | 97.3(3.7) | 100.0(0.0) | 97.3(3.7) | 0.99(0.02) |
| Pulmonic regurgitation | 96.4(2.5) | 96.4(2.5) | 100.0(0.0) | 0.98(0.01) | 98.5(2.0) | 98.5(2.0) | 100.0(0.0) | 0.99(0.01) | 95.6(3.9) | 96.3(2.6) | 99.2(1.7) | 0.98(0.02) | 98.5(2.0) | 98.5(2.0) | 100.0(0.0) | 0.99(0.01) |
| Pulmonic stenosis | 50.0(0.0) | 100.0(0.0) | 50.0(0.0) | 0.67(0.00) | 50.0(0.0) | 100.0(0.0) | 50.0(0.0) | 0.67(0.00) | 50.0(0.0) | 100.0(0.0) | 50.0(0.0) | 0.67(0.00) | 40.0(22.4) | 80.0(44.7) | 40.0(22.4) | 0.54(0.30) |
| RA pressure | 83.8(2.0) | 95.6(0.9) | 86.8(2.0) | 0.91(0.01) | 84.7(1.7) | 94.0(1.4) | 89.3(2.5) | 0.92(0.01) | 83.5(2.0) | 96.0(0.1) | 86.4(2.0) | 0.91(0.01) | 85.0(1.5) | 94.7(1.4) | 89.3(2.5) | 0.92(0.01) |
| RAE | 96.1(0.4) | 98.1(0.0) | 97.9(0.4) | 0.98(0.00) | 96.4(0.4) | 98.3(0.4) | 98.1(0.0) | 0.98(0.00) | 96.1(0.4) | 98.1(0.0) | 97.9(0.4) | 0.98(0.00) | 96.3(0.7) | 98.3(0.4) | 97.9(0.4) | 0.98(0.00) |
| RV function | 92.1(1.1) | 96.9(1.4) | 93.9(1.0) | 0.95(0.01) | 92.1(1.7) | 96.9(1.2) | 93.9(1.0) | 0.95(0.01) | 91.9(0.6) | 97.1(0.9) | 93.5(1.1) | 0.95(0.00) | 91.9(1.5) | 96.9(1.2) | 93.7(0.9) | 0.95(0.01) |
| RV size | 89.6(1.7) | 91.9(1.2) | 96.8(1.3) | 0.94(0.01) | 88.4(1.6) | 91.5(0.5) | 95.7(1.7) | 0.94(0.01) | 89.8(1.7) | 92.3(1.1) | 96.8(1.3) | 0.94(0.01) | 87.4(1.6) | 91.4(0.6) | 94.7(1.5) | 0.93(0.01) |
| RVSP | 96.9(0.8) | 97.1(0.9) | 98.6(0.5) | 0.98(0.01) | 96.7(0.5) | 97.4(0.9) | 98.6(0.5) | 0.98(0.00) | 96.9(0.8) | 97.1(0.9) | 98.6(0.5) | 0.98(0.01) | 96.6(0.7) | 97.4(0.9) | 98.5(0.8) | 0.98(0.00) |
| Tricuspid regurgitation | 98.6(0.7) | 99.7(0.4) | 98.7(0.6) | 0.99(0.00) | 98.6(0.5) | 99.1(0.3) | 99.3(0.5) | 0.99(0.00) | 98.3(0.8) | 99.7(0.4) | 98.4(0.8) | 0.99(0.01) | 98.4(0.6) | 99.4(0.3) | 99.0(0.4) | 0.99(0.00) |
| WMA | 91.9(0.8) | 97.3(0.4) | 94.3(0.6) | 0.96(0.00) | 90.7(1.0) | 96.1(1.3) | 93.9(0.4) | 0.95(0.01) | 90.8(1.4) | 97.3(0.4) | 93.1(1.3) | 0.95(0.01) | 90.7(0.7) | 96.4(1.2) | 93.6(0.3) | 0.95(0.00) |
| *All* | 95.7(0.2) | 97.6(0.2) | 97.4(0.1) | 0.98(0.00) | 95.7(0.1) | 97.6(0.2) | 97.5(0.3) | 0.98(0.00) | 95.4(0.2) | 97.7(0.2) | 97.1(0.2) | 0.97(0.00) | 95.5(0.2) | 97.7(0.2) | 97.2(0.2) | 0.97(0.00) |

^¥^ The performance of Prosthetic tricuspid valve, Prosthetic pulmonic valve and Tricuspid stenosis was not evaluated because of no confirmed cases.

Table S7. The performance of the fine-tuned BioclinicalBERT and BART-Large-CNN models at the rand seeds of 42, 736, 299, 971, 243 against the manual annotated results in the cardiac catheterization validation report dataset (n=300) at 0.1 and 0.5 cutoff thresholds of probability. The model was trained on 1584 reports with sequence length=512, batch size=4 and epochs=8.

| Extracted outcome^¥^ | BioclinicalBERT, Cutoff threshold of probability=0.1 | | | | | | | | | | | | | | | | | | | |
| --- | --- | --- | --- | --- | --- | --- | --- | --- | --- | --- | --- | --- | --- | --- | --- | --- | --- | --- | --- | --- |
|  | Accuracy (%) | | | | | Precision (%) | | | | | Recall (%) | | | | | F1score | | | | |
|  | Rand Seed=42 | Rand Seed=736 | Rand Seed=299 | Rand Seed=971 | Rand Seed=243 | Rand Seed=42 | Rand Seed=736 | Rand Seed=299 | Rand Seed=971 | Rand Seed=243 | Rand Seed=42 | Rand Seed=736 | Rand Seed=299 | Rand Seed=971 | Rand Seed=243 | Rand Seed=42 | Rand Seed=736 | Rand Seed=299 | Rand Seed=971 | Rand Seed=243 |
| Diagonal 1 | 90.08 | 93.8 | 89.55 | 93.08 | 91.60 | 95.16 | 97.58 | 93.75 | 96.03 | 96.77 | 91.47 | 93.80 | 93.02 | 93.80 | 93.02 | 0.93 | 0.96 | 0.93 | 0.95 | 0.95 |
| Diagonal 2 | 89.06 | 89.23 | 92.06 | 88.89 | 88.89 | 95.00 | 95.08 | 98.31 | 93.33 | 96.55 | 91.94 | 93.55 | 93.55 | 90.32 | 90.32 | 0.93 | 0.94 | 0.96 | 0.92 | 0.93 |
| Diagonal 3 | 75.00 | 80.00 | 75.00 | 80.00 | 75.00 | 92.31 | 100.00 | 85.71 | 100.00 | 92.31 | 80.00 | 80.00 | 80.00 | 80.00 | 80.00 | 0.86 | 0.89 | 0.83 | 0.89 | 0.86 |
| LAD | 94.12 | 94.14 | 95.50 | 95.16 | 95.85 | 95.77 | 95.12 | 96.17 | 96.15 | 96.52 | 94.12 | 94.46 | 95.50 | 95.16 | 95.85 | 0.95 | 0.95 | 0.96 | 0.96 | 0.96 |
| LCX | 94.03 | 94.78 | 93.38 | 94.40 | 93.68 | 94.74 | 95.13 | 94.07 | 94.76 | 94.38 | 95.82 | 96.58 | 96.58 | 96.20 | 95.82 | 0.95 | 0.96 | 0.95 | 0.95 | 0.95 |
| LIMA-LAD | 100.00 | 100.00 | 100.00 | 100.00 | 100.00 | 100.00 | 100.00 | 100.00 | 100.00 | 100.00 | 100.00 | 100.00 | 100.00 | 100.00 | 100.00 | 1.00 | 1.00 | 1.00 | 1.00 | 1.00 |
| Left Main | 99.30 | 99.65 | 99.30 | 99.65 | 99.65 | 99.30 | 100.00 | 99.30 | 99.65 | 99.65 | 99.30 | 99.65 | 99.30 | 99.65 | 99.65 | 0.99 | 1.00 | 0.99 | 1.00 | 1.00 |
| OM1 | 92.06 | 91.41 | 90.77 | 89.92 | 90.63 | 97.48 | 97.50 | 94.40 | 95.87 | 95.87 | 92.06 | 92.86 | 93.65 | 92.06 | 92.06 | 0.95 | 0.95 | 0.94 | 0.94 | 0.94 |
| OM2 | 75.47 | 78.85 | 79.59 | 81.63 | 82.00 | 78.43 | 83.67 | 84.78 | 88.89 | 87.23 | 90.91 | 93.18 | 88.64 | 90.91 | 93.18 | 0.84 | 0.88 | 0.87 | 0.90 | 0.90 |
| OM3 | 100.00 | 100.00 | 95.65 | 100.00 | 100.00 | 100.00 | 100.00 | 95.65 | 100.00 | 100.00 | 100.00 | 100.00 | 100.00 | 100.00 | 100.00 | 1.00 | 1.00 | 0.98 | 1.00 | 1.00 |
| OM4 | 100.00 | 33.33 | 100.00 | 100.00 | 50.00 | 100.00 | 33.33 | 100.00 | 100.00 | 50.00 | 100.00 | 100.00 | 100.00 | 100.00 | 100.00 | 1.00 | 0.50 | 1.00 | 1.00 | 0.67 |
| OM5 | 100.00 | 50.00 | 100.00 | 100.00 | 100.00 | 100.00 | 50.00 | 100.00 | 100.00 | 100.00 | 100.00 | 100.00 | 100.00 | 100.00 | 100.00 | 1.00 | 0.67 | 1.00 | 1.00 | 1.00 |
| PDA | 92.45 | 93.33 | 94.34 | 95.24 | 90.65 | 97.03 | 98.00 | 96.15 | 98.04 | 95.10 | 95.15 | 95.15 | 97.09 | 97.09 | 94.17 | 0.96 | 0.97 | 0.97 | 0.98 | 0.95 |
| PLV | 97.80 | 95.60 | 94.57 | 95.70 | 95.70 | 98.89 | 97.75 | 95.60 | 97.80 | 97.80 | 97.80 | 95.60 | 95.60 | 97.80 | 97.80 | 0.98 | 0.97 | 0.96 | 0.98 | 0.98 |
| RCA | 96.03 | 94.96 | 94.64 | 96.42 | 95.36 | 97.44 | 96.00 | 94.98 | 96.76 | 96.04 | 96.03 | 95.31 | 95.67 | 97.11 | 96.39 | 0.97 | 0.96 | 0.95 | 0.97 | 0.96 |
| Ramus | 97.67 | 95.35 | 97.67 | 95.35 | 97.67 | 100.00 | 100.00 | 100.00 | 100.00 | 100.00 | 97.67 | 95.35 | 97.67 | 95.35 | 97.67 | 0.99 | 0.98 | 0.99 | 0.98 | 0.99 |
| SVG-D1 | 100.00 | 100.00 | 100.00 | 100.00 | 100.00 | 100.00 | 100.00 | 100.00 | 100.00 | 100.00 | 100.00 | 100.00 | 100.00 | 100.00 | 100.00 | 1.00 | 1.00 | 1.00 | 1.00 | 1.00 |
| SVG-OM1 | 100.00 | 100.00 | 100.00 | 100.00 | 100.00 | 100.00 | 100.00 | 100.00 | 100.00 | 100.00 | 100.00 | 100.00 | 100.00 | 100.00 | 100.00 | 1.00 | 1.00 | 1.00 | 1.00 | 1.00 |
| SVG-PDA | 100.00 | 100.00 | 100.00 | 100.00 | 100.00 | 100.00 | 100.00 | 100.00 | 100.00 | 100.00 | 100.00 | 100.00 | 100.00 | 100.00 | 100.00 | 1.00 | 1.00 | 1.00 | 1.00 | 1.00 |
| *ALL* | 94.16 | 94.28 | 94.08 | 94.83 | 94.29 | 96.38 | 96.44 | 95.63 | 96.68 | 96.39 | 95.34 | 95.62 | 95.85 | 95.96 | 95.79 | 0.96 | 0.96 | 0.96 | 0.96 | 0.96 |
|  | BioclinicalBERT, Cutoff threshold of probability=0.5 | | | | | | | | | | | | | | | | | | | |
|  | Accuracy (%) | | | | | Precision (%) | | | | | Recall (%) | | | | | F1score | | | | |
|  | Rand Seed=42 | Rand Seed=736 | Rand Seed=299 | Rand Seed=971 | Rand Seed=243 | Rand Seed=42 | Rand Seed=736 | Rand Seed=299 | Rand Seed=971 | Rand Seed=243 | Rand Seed=42 | Rand Seed=736 | Rand Seed=299 | Rand Seed=971 | Rand Seed=243 | Rand Seed=42 | Rand Seed=736 | Rand Seed=299 | Rand Seed=971 | Rand Seed=243 |
| Diagonal 1 | 89.31 | 93.02 | 89.55 | 90.77 | 91.60 | 95.12 | 97.56 | 93.75 | 95.93 | 96.77 | 90.70 | 93.02 | 93.02 | 91.47 | 93.02 | 0.93 | 0.95 | 0.93 | 0.94 | 0.95 |
| Diagonal 2 | 89.06 | 90.63 | 90.48 | 85.71 | 87.30 | 96.61 | 96.67 | 98.28 | 96.43 | 96.49 | 91.94 | 93.55 | 91.94 | 87.10 | 88.71 | 0.94 | 0.95 | 0.95 | 0.92 | 0.92 |
| Diagonal 3 | 75.00 | 80.00 | 75.00 | 80.00 | 75.00 | 92.31 | 100.00 | 85.71 | 100.00 | 92.31 | 80.00 | 80.00 | 80.00 | 80.00 | 80.00 | 0.86 | 0.89 | 0.83 | 0.89 | 0.86 |
| LAD | 93.43 | 94.14 | 94.81 | 93.43 | 95.85 | 95.74 | 95.12 | 96.14 | 96.43 | 96.52 | 93.43 | 94.46 | 94.81 | 93.43 | 95.85 | 0.95 | 0.95 | 0.95 | 0.95 | 0.96 |
| LCX | 94.01 | 94.78 | 93.01 | 92.51 | 94.03 | 95.08 | 95.49 | 94.05 | 95.00 | 94.74 | 95.44 | 96.58 | 96.20 | 93.92 | 95.82 | 0.95 | 0.96 | 0.95 | 0.94 | 0.95 |
| LIMA-LAD | 100.00 | 100.00 | 100.00 | 100.00 | 100.00 | 100.00 | 100.00 | 100.00 | 100.00 | 100.00 | 100.00 | 100.00 | 100.00 | 100.00 | 100.00 | 1.00 | 1.00 | 1.00 | 1.00 | 1.00 |
| Left Main | 99.30 | 99.30 | 99.30 | 96.14 | 99.65 | 99.30 | 100.00 | 99.30 | 99.64 | 99.65 | 99.30 | 99.30 | 99.30 | 96.14 | 99.65 | 0.99 | 1.00 | 0.99 | 0.98 | 1.00 |
| OM1 | 92.06 | 90.63 | 90.77 | 88.37 | 90.63 | 97.48 | 97.48 | 94.40 | 95.80 | 95.87 | 92.06 | 92.06 | 93.65 | 90.48 | 92.06 | 0.95 | 0.95 | 0.94 | 0.93 | 0.94 |
| OM2 | 73.58 | 83.33 | 79.59 | 79.59 | 82.00 | 78.00 | 90.91 | 84.78 | 88.64 | 87.23 | 88.64 | 90.91 | 88.64 | 88.64 | 93.18 | 0.83 | 0.91 | 0.87 | 0.89 | 0.9 |
| OM3 | 100.00 | 100.00 | 95.65 | 100.00 | 100.00 | 100.00 | 100.00 | 95.65 | 100.00 | 100.00 | 100.00 | 100.00 | 100.00 | 100.00 | 100.00 | 1.00 | 1.00 | 0.98 | 1.00 | 1.00 |
| OM4 | 100.00 | 50.00 | 100.00 | 100.00 | 50.00 | 100.00 | 50.00 | 100.00 | 100.00 | 50.00 | 100.00 | 100.00 | 100.00 | 100.00 | 100.00 | 1.00 | 0.67 | 1.00 | 1.00 | 0.67 |
| OM5 | 100.00 | 100.00 | 100.00 | 100.00 | 100.00 | 100.00 | 100.00 | 100.00 | 100.00 | 100.00 | 100.00 | 100.00 | 100.00 | 100.00 | 100.00 | 1.00 | 1.00 | 1.00 | 1.00 | 1.00 |
| PDA | 92.45 | 93.33 | 94.34 | 91.43 | 90.65 | 97.03 | 98.00 | 96.15 | 97.96 | 95.10 | 95.15 | 95.15 | 97.09 | 93.20 | 94.17 | 0.96 | 0.97 | 0.97 | 0.96 | 0.95 |
| PLV | 97.80 | 95.60 | 94.57 | 93.55 | 95.70 | 98.89 | 98.86 | 96.67 | 97.75 | 97.80 | 97.80 | 95.60 | 95.60 | 95.60 | 97.80 | 0.98 | 0.97 | 0.96 | 0.97 | 0.98 |
| RCA | 95.31 | 94.24 | 94.29 | 94.62 | 95.00 | 97.42 | 95.97 | 95.65 | 96.70 | 96.38 | 95.31 | 94.58 | 95.31 | 95.31 | 96.03 | 0.96 | 0.95 | 0.95 | 0.96 | 0.96 |
| Ramus | 97.67 | 95.35 | 97.67 | 93.02 | 97.67 | 100.00 | 100.00 | 100.00 | 100.00 | 100.00 | 97.67 | 95.35 | 97.67 | 93.02 | 97.67 | 0.99 | 0.98 | 0.99 | 0.96 | 0.99 |
| SVG-D1 | 100.00 | 100.00 | 100.00 | 100.00 | 100.00 | 100.00 | 100.00 | 100.00 | 100.00 | 100.00 | 100.00 | 100.00 | 100.00 | 100.00 | 100.00 | 1.00 | 1.00 | 1.00 | 1.00 | 1.00 |
| SVG-OM1 | 50.00 | 100.00 | 100.00 | 100.00 | 100.00 | 100.00 | 100.00 | 100.00 | 100.00 | 100.00 | 50.00 | 100.00 | 100.00 | 100.00 | 100.00 | 0.67 | 1.00 | 1.00 | 1.00 | 1.00 |
| SVG-PDA | 100.00 | 100.00 | 100.00 | 100.00 | 100.00 | 100.00 | 100.00 | 100.00 | 100.00 | 100.00 | 100.00 | 100.00 | 100.00 | 100.00 | 100.00 | 1.00 | 1.00 | 1.00 | 1.00 | 1.00 |
| *ALL* | 93.76 | 94.31 | 93.8 | 92.58 | 94.23 | 96.47 | 96.93 | 95.78 | 96.82 | 96.50 | 94.88 | 95.28 | 95.56 | 93.63 | 95.68 | 0.96 | 0.96 | 0.96 | 0.95 | 0.96 |
|  | BART-Large-CNN, Cutoff threshold of probability=0.1 | | | | | | | | | | | | | | | | | | | |
|  | Accuracy (%) | | | | | Precision (%) | | | | | Recall (%) | | | | | F1score | | | | |
|  | Rand Seed=42 | Rand Seed=736 | Rand Seed=299 | Rand Seed=971 | Rand Seed=243 | Rand Seed=42 | Rand Seed=736 | Rand Seed=299 | Rand Seed=971 | Rand Seed=243 | Rand Seed=42 | Rand Seed=736 | Rand Seed=299 | Rand Seed=971 | Rand Seed=243 | Rand Seed=42 | Rand Seed=736 | Rand Seed=299 | Rand Seed=971 | Rand Seed=243 |
| Diagonal 1 | 90.00 | 91.60 | 88.64 | 87.12 | 90.84 | 94.35 | 95.24 | 92.13 | 92.00 | 92.97 | 90.70 | 93.02 | 90.70 | 89.15 | 92.25 | 0.92 | 0.94 | 0.91 | 0.91 | 0.93 |
| Diagonal 2 | 89.06 | 90.63 | 92.06 | 93.55 | 90.48 | 95.00 | 95.08 | 96.67 | 98.31 | 95.00 | 91.94 | 93.55 | 93.55 | 93.55 | 91.94 | 0.93 | 0.94 | 0.95 | 0.96 | 0.93 |
| Diagonal 3 | 73.33 | 80.00 | 80.00 | 86.67 | 80.00 | 100.00 | 100.00 | 100.00 | 100.00 | 92.31 | 73.33 | 80.00 | 80.00 | 86.67 | 80.00 | 0.85 | 0.89 | 0.89 | 0.93 | 0.86 |
| LAD | 95.85 | 95.16 | 93.77 | 95.16 | 94.81 | 96.85 | 96.15 | 94.76 | 96.15 | 95.47 | 95.85 | 95.16 | 93.77 | 95.16 | 94.81 | 0.96 | 0.96 | 0.94 | 0.96 | 0.95 |
| LCX | 95.90 | 94.46 | 95.56 | 96.30 | 95.91 | 95.90 | 94.46 | 95.56 | 96.30 | 96.27 | 97.72 | 97.34 | 98.10 | 98.86 | 98.10 | 0.97 | 0.96 | 0.97 | 0.98 | 0.97 |
| LIMA-LAD | 100.00 | 100.00 | 100.00 | 100.00 | 100.00 | 100.00 | 100.00 | 100.00 | 100.00 | 100.00 | 100.00 | 100.00 | 100.00 | 100.00 | 100.00 | 1.00 | 1.00 | 1.00 | 1.00 | 1.00 |
| Left Main | 99.65 | 99.30 | 99.30 | 99.30 | 99.65 | 99.65 | 99.30 | 99.65 | 99.65 | 99.65 | 99.65 | 99.65 | 99.30 | 99.30 | 99.65 | 1.00 | 0.99 | 0.99 | 0.99 | 1.00 |
| OM1 | 92.06 | 92.13 | 91.27 | 92.25 | 92.19 | 99.15 | 96.69 | 97.46 | 95.97 | 96.72 | 92.06 | 92.86 | 91.27 | 94.44 | 93.65 | 0.95 | 0.95 | 0.94 | 0.95 | 0.95 |
| OM2 | 95.65 | 85.11 | 82.00 | 85.42 | 85.42 | 95.65 | 90.91 | 85.42 | 89.13 | 89.13 | 100.00 | 90.91 | 93.18 | 93.18 | 93.18 | 0.98 | 0.91 | 0.89 | 0.91 | 0.91 |
| OM3 | 100.00 | 100.00 | 95.65 | 95.45 | 100.00 | 100.00 | 100.00 | 95.65 | 100.00 | 100.00 | 100.00 | 100.00 | 100.00 | 95.45 | 100.00 | 1.00 | 1.00 | 0.98 | 0.98 | 1.00 |
| OM4 | 50.00 | 100.00 | 100.00 | 33.33 | 20.00 | 50.00 | 100.00 | 100.00 | 33.33 | 20.00 | 100.00 | 100.00 | 100.00 | 100.00 | 100.00 | 0.67 | 1.00 | 1.00 | 0.5 | 0.33 |
| OM5 | 50.00 | 100.00 | 100.00 | 100.00 | 100.00 | 50.00 | 100.00 | 100.00 | 100.00 | 100.00 | 100.00 | 100.00 | 100.00 | 100.00 | 100.00 | 0.67 | 1.00 | 1.00 | 1.00 | 1.00 |
| PDA | 90.38 | 95.19 | 93.33 | 88.79 | 93.27 | 98.95 | 99.00 | 98.00 | 95.00 | 98.98 | 91.26 | 96.12 | 95.15 | 92.23 | 94.17 | 0.95 | 0.98 | 0.97 | 0.94 | 0.97 |
| PLV | 95.65 | 96.77 | 94.62 | 94.68 | 95.70 | 96.70 | 96.77 | 96.70 | 95.70 | 97.80 | 96.70 | 98.90 | 96.70 | 97.80 | 97.80 | 0.97 | 0.98 | 0.97 | 0.97 | 0.98 |
| RCA | 97.12 | 96.4 | 96.76 | 96.40 | 97.84 | 97.12 | 96.75 | 96.76 | 97.45 | 98.19 | 97.47 | 96.75 | 97.11 | 96.75 | 98.19 | 0.97 | 0.97 | 0.97 | 0.97 | 0.98 |
| Ramus | 97.67 | 97.67 | 100.00 | 100.00 | 97.67 | 100.00 | 100.00 | 100.00 | 100.00 | 100.00 | 97.67 | 97.67 | 100.00 | 100.00 | 97.67 | 0.99 | 0.99 | 1.00 | 1.00 | 0.99 |
| SVG-D1 | 100.00 | 100.00 | 100.00 | 100.00 | 100.00 | 100.00 | 100.00 | 100.00 | 100.00 | 100.00 | 100.00 | 100.00 | 100.00 | 100.00 | 100.00 | 1.00 | 1.00 | 1.00 | 1.00 | 1.00 |
| SVG-OM1 | 100.00 | 100.00 | 100.00 | 100.00 | 100.00 | 100.00 | 100.00 | 100.00 | 100.00 | 100.00 | 100.00 | 100.00 | 100.00 | 100.00 | 100.00 | 1.00 | 1.00 | 1.00 | 1.00 | 1.00 |
| SVG-PDA | 100.00 | 100.00 | 100.00 | 100.00 | 100.00 | 100.00 | 100.00 | 100.00 | 100.00 | 100.00 | 100.00 | 100.00 | 100.00 | 100.00 | 100.00 | 1.00 | 1.00 | 1.00 | 1.00 | 1.00 |
| *ALL* | 95.21 | 95.11 | 94.55 | 94.62 | 95.12 | 97.24 | 96.69 | 96.28 | 96.51 | 96.69 | 96.02 | 96.25 | 95.79 | 96.08 | 96.36 | 0.97 | 0.96 | 0.96 | 0.96 | 0.97 |
|  | BART-Large-CNN, Cutoff threshold of probability=0.5 | | | | | | | | | | | | | | | | | | | |
|  | Accuracy (%) | | | | | Precision (%) | | | | | Recall (%) | | | | | F1score | | | | |
|  | Rand Seed=42 | Rand Seed=736 | Rand Seed=299 | Rand Seed=971 | Rand Seed=243 | Rand Seed=42 | Rand Seed=736 | Rand Seed=299 | Rand Seed=971 | Rand Seed=243 | Rand Seed=42 | Rand Seed=736 | Rand Seed=299 | Rand Seed=971 | Rand Seed=243 | Rand Seed=42 | Rand Seed=736 | Rand Seed=299 | Rand Seed=971 | Rand Seed=243 |
| Diagonal 1 | 90.00 | 91.6 | 89.31 | 87.12 | 90.84 | 94.35 | 95.24 | 92.86 | 92.00 | 92.97 | 90.70 | 93.02 | 90.7 | 89.15 | 92.25 | 0.92 | 0.94 | 0.92 | 0.91 | 0.93 |
| Diagonal 2 | 89.06 | 90.63 | 92.06 | 93.55 | 90.48 | 95.00 | 95.08 | 96.67 | 100.00 | 95.00 | 91.94 | 93.55 | 93.55 | 93.55 | 91.94 | 0.93 | 0.94 | 0.95 | 0.97 | 0.93 |
| Diagonal 3 | 73.33 | 80.00 | 80.00 | 86.67 | 80.00 | 100.00 | 100.00 | 100.00 | 100.00 | 92.31 | 73.33 | 80.00 | 80.00 | 86.67 | 80.00 | 0.85 | 0.89 | 0.89 | 0.93 | 0.86 |
| LAD | 95.50 | 95.16 | 93.77 | 94.46 | 94.46 | 96.84 | 96.15 | 95.09 | 96.13 | 95.45 | 95.50 | 95.16 | 93.77 | 94.46 | 94.46 | 0.96 | 0.96 | 0.94 | 0.95 | 0.95 |
| LCX | 95.90 | 94.46 | 95.54 | 96.30 | 96.27 | 95.90 | 94.46 | 95.90 | 96.30 | 96.63 | 97.72 | 97.34 | 97.72 | 98.86 | 98.10 | 0.97 | 0.96 | 0.97 | 0.98 | 0.97 |
| LIMA-LAD | 100.00 | 100.00 | 100.00 | 100.00 | 100.00 | 100.00 | 100.00 | 100.00 | 100.00 | 100.00 | 100.00 | 100.00 | 100.00 | 100.00 | 100.00 | 1.00 | 1.00 | 1.00 | 1.00 | 1.00 |
| Left Main | 99.65 | 99.30 | 99.30 | 99.30 | 99.65 | 99.65 | 99.30 | 99.65 | 99.65 | 99.65 | 99.65 | 99.65 | 99.30 | 99.30 | 99.65 | 1.00 | 0.99 | 0.99 | 0.99 | 1.00 |
| OM1 | 91.27 | 92.13 | 91.27 | 92.25 | 92.19 | 99.14 | 96.69 | 97.46 | 95.97 | 96.72 | 91.27 | 92.86 | 91.27 | 94.44 | 93.65 | 0.95 | 0.95 | 0.94 | 0.95 | 0.95 |
| OM2 | 95.65 | 86.96 | 83.67 | 85.42 | 87.23 | 95.65 | 93.02 | 87.23 | 89.13 | 91.11 | 100.00 | 90.91 | 93.18 | 93.18 | 93.18 | 0.98 | 0.92 | 0.9 | 0.91 | 0.92 |
| OM3 | 100.00 | 100.00 | 100.00 | 95.45 | 100.00 | 100.00 | 100.00 | 100.00 | 100.00 | 100.00 | 100.00 | 100.00 | 100.00 | 95.45 | 100.00 | 1.00 | 1.00 | 1.00 | 0.98 | 1.00 |
| OM4 | 50.00 | 100.00 | 100.00 | 50.00 | 33.33 | 50.00 | 100.00 | 100.00 | 50.00 | 33.33 | 100.00 | 100.00 | 100.00 | 100.00 | 100.00 | 0.67 | 1.00 | 1.00 | 0.67 | 0.50 |
| OM5 | 50.00 | 100.00 | 100.00 | 100.00 | 100.00 | 50.00 | 100.00 | 100.00 | 100.00 | 100.00 | 100.00 | 100.00 | 100.00 | 100.00 | 100.00 | 0.67 | 1.00 | 1.00 | 1.00 | 1.00 |
| PDA | 90.38 | 95.19 | 93.33 | 88.79 | 93.27 | 98.95 | 99.00 | 98.00 | 95.00 | 98.98 | 91.26 | 96.12 | 95.15 | 92.23 | 94.17 | 0.95 | 0.98 | 0.97 | 0.94 | 0.97 |
| PLV | 95.65 | 96.77 | 94.62 | 93.62 | 94.62 | 96.70 | 96.77 | 96.70 | 95.65 | 97.78 | 96.70 | 98.90 | 96.70 | 96.70 | 96.70 | 0.97 | 0.98 | 0.97 | 0.96 | 0.97 |
| RCA | 97.12 | 96.40 | 96.40 | 96.40 | 98.19 | 97.12 | 96.75 | 96.75 | 97.45 | 98.55 | 97.47 | 96.75 | 96.75 | 96.75 | 98.19 | 0.97 | 0.97 | 0.97 | 0.97 | 0.98 |
| Ramus | 97.67 | 97.67 | 100.00 | 100.00 | 97.67 | 100.00 | 100.00 | 100.00 | 100.00 | 100.00 | 97.67 | 97.67 | 100.00 | 100.00 | 97.67 | 0.99 | 0.99 | 1.00 | 1.00 | 0.99 |
| SVG-D1 | 100.00 | 100.00 | 100.00 | 100.00 | 100.00 | 100.00 | 100.00 | 100.00 | 100.00 | 100.00 | 100.00 | 100.00 | 100.00 | 100.00 | 100.00 | 1.00 | 1.00 | 1.00 | 1.00 | 1.00 |
| SVG-OM1 | 100.00 | 100.00 | 100.00 | 100.00 | 100.00 | 100.00 | 100.00 | 100.00 | 100.00 | 100.00 | 100.00 | 100.00 | 100.00 | 100.00 | 100.00 | 1.00 | 1.00 | 1.00 | 1.00 | 1.00 |
| SVG-PDA | 100.00 | 100.00 | 100.00 | 100.00 | 100.00 | 100.00 | 100.00 | 100.00 | 100.00 | 100.00 | 100.00 | 100.00 | 100.00 | 100.00 | 100.00 | 1.00 | 1.00 | 1.00 | 1.00 | 1.00 |
| *ALL* | 95.09 | 95.16 | 94.65 | 94.51 | 95.27 | 97.23 | 96.74 | 96.56 | 96.62 | 96.96 | 95.90 | 96.25 | 95.68 | 95.90 | 96.25 | 0.97 | 0.96 | 0.96 | 0.96 | 0.97 |

^¥^ The performance of the outcome categories in supplemental Table S2, but not listed here was not evaluated because of no confirmed cases.

Table S8. The mean and standard deviation of the performance of the fine-tuned BioclinicalBERT and BART-Large-CNN models at five different rand seeds of 42, 736, 299, 971, 243 against the manual annotated results in the cardiac catheterization validation report dataset (n=300) at 0.1 and 0.5 cutoff thresholds of probability. The model was trained on 1584 reports with sequence length=512, batch size=4 and epochs=8.

| Extracted outcome^¥^ | Cutoff threshold of probability=0.1 | | | | | | | | Cutoff threshold of probability=0.5 | | | | | | | |
| --- | --- | --- | --- | --- | --- | --- | --- | --- | --- | --- | --- | --- | --- | --- | --- | --- |
|  | BioclinicalBERT | | | | BART-Large-CNN | | | | BioclinicalBERT | | | | BART-Large-CNN | | | |
|  | Accuracy (%) | Precision (%) | Recall (%) | F1score | Accuracy (%) | Precision (%) | Recall (%) | F1score | Accuracy (%) | Precision (%) | Recall (%) | F1score | Accuracy (%) | Precision (%) | Recall (%) | F1score |
| Diagonal 1 | 91.6(1.8) | 95.9(1.5) | 93.0(1.0) | 0.94(0.01) | 89.6(1.8) | 93.3(1.4) | 91.2(1.5) | 0.92(0.01) | 90.9(1.5) | 95.8(1.5) | 92.3(1.1) | 0.94(0.01) | 89.8(1.7) | 93.5(1.3) | 91.2(1.5) | 0.92(0.01) |
| Diagonal 2 | 89.6(1.4) | 95.7(1.9) | 91.9(1.6) | 0.94(0.01) | 91.2(1.7) | 96.0(1.5) | 92.9(0.9) | 0.94(0.01) | 88.6(2.1) | 96.9(0.8) | 90.7(2.7) | 0.94(0.02) | 91.2(1.7) | 96.4(2.2) | 92.9(0.9) | 0.95(0.01) |
| Diagonal 3 | 77.0(2.7) | 94.1(6.1) | 80.0(0.0) | 0.86(0.03) | 80.0(4.7) | 98.5(3.4) | 80.0(4.7) | 0.88(0.03) | 77.0(2.7) | 94.1(6.1) | 80.0(0.0) | 0.86(0.03) | 80.0(4.7) | 98.5(3.4) | 80.0(4.7) | 0.88(0.03) |
| LAD | 95.0(0.8) | 96.0(0.5) | 95.0(0.7) | 0.95(0.01) | 95.0(0.8) | 95.9(0.8) | 95.0(0.8) | 0.95(0.01) | 94.3(1.0) | 96.0(0.6) | 94.4(1.0) | 0.95(0.01) | 94.7(0.7) | 95.9(0.7) | 94.7(0.7) | 0.95(0.01) |
| LCX | 94.1(0.6) | 94.6(0.4) | 96.2(0.4) | 0.95(0.00) | 95.6(0.7) | 95.7(0.8) | 98.0(0.6) | 0.97(0.01) | 93.7(0.9) | 94.9(0.5) | 95.6(1.0) | 0.95(0.01) | 95.7(0.8) | 95.8(0.8) | 98.0(0.6) | 0.97(0.01) |
| LIMA-LAD | 100.0(0.0) | 100.0(0.0) | 100.0(0.0) | 1.00(0.00) | 100.0(0.0) | 100.0(0.0) | 100.0(0.0) | 1.00(0.00) | 100.0(0.0) | 100.0(0.0) | 100.0(0.0) | 1.00(0.00) | 100.0(0.0) | 100.0(0.0) | 100.0(0.0) | 1.00(0.00) |
| Left Main | 99.5(0.2) | 99.6(0.3) | 99.5(0.2) | 1.00(0.00) | 99.4(0.2) | 99.6(0.2) | 99.5(0.2) | 1.00(0.00) | 98.7(1.5) | 99.6(0.3) | 98.7(1.5) | 0.99(0.01) | 99.4(0.2) | 99.6(0.2) | 99.5(0.2) | 1.00(0.00) |
| OM1 | 91.0(0.8) | 96.2(1.3) | 92.5(0.7) | 0.94(0.01) | 92.0(0.4) | 97.2(1.2) | 92.9(1.3) | 0.95(0.00) | 90.5(1.3) | 96.2(1.3) | 92.1(1.1) | 0.94(0.01) | 91.8(0.5) | 97.2(1.2) | 92.7(1.4) | 0.95(0.00) |
| OM2 | 79.5(2.6) | 84.6(4.0) | 91.4(1.9) | 0.88(0.02) | 86.7(5.2) | 90.1(3.7) | 94.1(3.5) | 0.92(0.03) | 79.6(3.7) | 85.9(5.0) | 90.0(2.0) | 0.88(0.03) | 87.8(4.6) | 91.2(3.3) | 94.1(3.5) | 0.93(0.03) |
| OM3 | 99.1(1.9) | 99.1(1.9) | 100.0(0.0) | 1.00(0.01) | 98.2(2.4) | 99.1(1.9) | 99.1(2.0) | 0.99(0.01) | 99.1(1.9) | 99.1(1.9) | 100.0(0.0) | 1.00(0.01) | 99.1(2.0) | 100.0(0.0) | 99.1(2.0) | 1.00(0.01) |
| OM4 | 76.7(32.5) | 76.7(32.5) | 100.0(0.0) | 0.83(0.24) | 60.7(37.5) | 60.7(37.5) | 100.0(0.0) | 0.70(0.30) | 80.0(27.4) | 80.0(27.4) | 100.0(0.0) | 0.87(0.18) | 66.7(31.2) | 66.7(31.2) | 100.0(0.0) | 0.77(0.22) |
| OM5 | 90.0(22.4) | 90(22.4) | 100.0(0.0) | 0.93(0.15) | 90.0(22.4) | 90.0(22.4) | 100.0(0.0) | 0.93(0.15) | 100.0(0.0) | 100.0(0.0) | 100.0(0.0) | 1.00(0.00) | 90.0(22.4) | 90.0(22.4) | 100.0(0.0) | 0.93(0.15) |
| PDA | 93.2(1.8) | 96.9(1.3) | 95.7(1.3) | 0.96(0.01) | 92.2(2.6) | 98.0(1.7) | 93.8(2.0) | 0.96(0.02) | 92.4(1.5) | 96.9(1.2) | 95.0(1.4) | 0.96(0.01) | 92.2(2.6) | 98.0(1.7) | 93.8(2.0) | 0.96(0.02) |
| PLV | 95.9(1.2) | 97.6(1.2) | 96.9(1.2) | 0.97(0.01) | 95.5(0.9) | 96.7(0.7) | 97.6(0.9) | 0.97(0.01) | 95.4(1.56) | 98.0(0.9) | 96.5(1.2) | 0.97(0.01) | 95.1(1.2) | 96.7(0.8) | 97.1(1.0) | 0.97(0.01) |
| RCA | 95.5(0.7) | 96.2(0.9) | 96.1(0.7) | 0.96(0.01) | 96.9(0.6) | 97.3(0.6) | 97.3(0.6) | 0.97(0.01) | 94.7(0.5) | 96.4(0.7) | 95.3(0.5) | 0.96(0.00) | 96.9(0.8) | 97.3(0.7) | 97.2(0.7) | 0.97(0.01) |
| Ramus | 96.7(1.23) | 100.0(0.0) | 96.7(1.3) | 0.98(0.01) | 98.6(1.3) | 100.0(0.0) | 98.6(1.3) | 0.99(0.01) | 96.3(2.1) | 100.0(0.0) | 96.3(2.1) | 0.98(0.01) | 98.6(1.3) | 100.0(0.0) | 98.6(1.3) | 0.99(0.01) |
| SVG-D1 | 100.0(0.0) | 100.0(0.0) | 100.0(0.0) | 1.00(0.00) | 100.0(0.0) | 100.0(0.0) | 100.0(0.0) | 1.00(0.00) | 100.0(0.0) | 100.0(0.0) | 100.0(0.0) | 1.00(0.00) | 100.0(0.0) | 100.0(0.0) | 100.0(0.0) | 1.00(0.00) |
| SVG-OM1 | 100.0(0.0) | 100.0(0.0) | 100.0(0.0) | 1.00(0.00) | 100.0(0.0) | 100.0(0.0) | 100.0(0.0) | 1.00(0.00) | 90.0(22.4) | 100.0(0.0) | 90.0(22.4) | 0.93(0.15) | 100.0(0.0) | 100.0(0.0) | 100.0(0.0) | 1.00(0.00) |
| SVG-PDA | 100.0(0.0) | 100.0(0.0) | 100.0(0.0) | 1.00(0.00) | 100.0(0.0) | 100.0(0.0) | 100.0(0.0) | 1.00(0.00) | 100.0(0.0) | 100.0(0.0) | 100.0(0.0) | 1.00(0.00) | 100.0(0.0) | 100.0(0.0) | 100.0(0.0) | 1.00(0.00) |
| *ALL* | 94.3(0.2) | 96.3(0.4) | 95.7(0.2) | 0.96(0.00) | 94.9(0.3) | 96.7(0.4) | 96.1(0.2) | 0.96(0.00) | 93.7(0.7) | 96.5(0.5) | 95.0(0.8) | 0.96(0.00) | 94.9(0.3) | 96.8(0.3) | 96.0(0.3) | 0.96(0.00) |

^¥^ The performance of the outcome categories in supplemental Table S2, but not listed here was not evaluated because of no confirmed cases.

Table S9. Summary of discrepancy at 0.1 cutoff threshold of predicted probability by the model against the manual annotated results from the validation datasets.

| **Report group** | **Model** | **Type of discrepancy** | **Number of cases** | **Mismatched details** | |
| --- | --- | --- | --- | --- | --- |
|  |  |  |  | **Extracted by model** | **Extracted by annotator** |
| Echocardiography | BioclinicalBERT | Model and manual identified different values | 3 | LV size: normal | LV size: small |
|  |  |  | 2 | RVSP: 55; 144 | RVSP: 20; normal |
|  |  |  | 1 | Mitral Regurgitation: minimal | Mitral Regurgitation: mild |
|  |  |  | 1 | Aortic Stenosis: Aortic Stenosis | Aortic Stenosis: with evidence |
|  |  |  | 1 | Mitral Stenosis: Mitral Stenosis | Aortic Stenosis: with evidence |
|  |  |  | 1 | LVH: moderate | LVH: mild |
|  |  |  | 1 | Diastolic Dysfunction: moderate | Diastolic dysfunction: normal |
|  |  |  | 1 | LVEF: 65% | LVEF: 35% |
|  |  |  | 1 | Pericardial Effusion: small | Pericardial Effusion: moderate |
|  |  |  | 1 | RV Function: severely reduced | RV Function: normal |
|  |  | Model extracted values not described in report | 3 | Aortic Regurgitation: mild (1); one mild to moderate (1); trace (1) | No extracted values |
|  |  |  | 2 | Aortic Stenosis: severe | No extracted values |
|  |  |  | 1 | Diastolic Dysfunction: normal | No extracted values |
|  |  |  | 2 | LAE: enlarged (1); dilated (1) | No extracted values |
|  |  |  | 1 | LVEF: normal | No extracted values |
|  |  |  | 2 | LVH: normal | No extracted values |
|  |  |  | 2 | LV Size: normal | No extracted values |
|  |  |  | 1 | Mitral Regurgitation: without | No extracted values |
|  |  |  | 1 | Mitral Stenosis: without | No extracted values |
|  |  |  | 3 | Pericardial Effusion: small (2); pericardial effusion (1) | No extracted values |
|  |  |  | 2 | Pulmonic Regurgitation: no | No extracted values |
|  |  |  | 2 | RAE: enlarged (1); moderate (1) | No extracted values |
|  |  |  | 2 | RA Pressure: 3mmHg (1); 5mmHg (1) | No extracted values |
|  |  |  | 1 | RV Function: normal | No extracted values |
|  |  |  | 8 | RV size: normal (7); increased (1) | No extracted values |
|  |  |  | 2 | RVSP: 35 (1); 47 (1) | No extracted values |
|  |  |  | 3 | WMA: preserved (1); septal bounce (1); dyskinesis of the anteroseptum (1) | No extracted values |
|  |  | Model failed to correct label values described in report | 20 | Model extracted completely different values with probability score < 0.1 | |
|  |  |  | 26 | Model extracted same annotated values but with probability score < 0.1 | |
|  | BART-Large-CNN | Model and manual identified different values | 2 | LVEF: grade I; moderate | LVEF: normal; 40% |
|  |  |  | 1 | LV Size: normal | LV Size: small |
|  |  |  | 1 | Tricuspid Regurgitation: tr | Tricuspid Regurgitation: mild |
|  |  |  | 1 | RVSP: 55 | RVSP: 22 |
|  |  |  | 1 | Diastolic Dysfunction: moderate | Diastolic Dysfunction: normal |
|  |  |  | 1 | RA Pressure: 20 | RA Pressure: 15 |
|  |  |  | 1 | Pericardial Effusion: small | Pericardial Effusion: moderate |
|  |  |  | 1 | RV Function: Severely reduced | RV Function: normal |
|  |  | Model extracted values not described in report | 3 | Aortic Regurgitation: mild (1); one mild to moderate (1); trace (1) | No extracted values |
|  |  |  | 2 | Aortic Stenosis: severe | No extracted values |
|  |  |  | 1 | Diastolic Dysfunction: normal | No extracted values |
|  |  |  | 3 | LAE: enlarged (1); dilated (1); LAE (1) | No extracted values |
|  |  |  | 1 | LVEF: normal | No extracted values |
|  |  |  | 2 | LVH: normal | No extracted values |
|  |  |  | 1 | LV Size: normal | No extracted values |
|  |  |  | 1 | Mitral Regurgitation: without | No extracted values |
|  |  |  | 1 | Mitral Stenosis: without | No extracted values |
|  |  |  | 2 | Pericardial Effusion: small (1); present (1) | No extracted values |
|  |  |  | 1 | Prosthetic Aortic Valve: transcutaneously inserted aortic valve | No extracted values |
|  |  |  | 1 | Pulmonic Regurgitation: no | No extracted values |
|  |  |  | 1 | RAE: enlarged | No extracted values |
|  |  |  | 2 | RA Pressure: 3 (1); 5 (1) | No extracted values |
|  |  |  | 1 | RV Function: systolic dysfunction | No extracted values |
|  |  |  | 8 | RV Size: normal (7); increased (1) | No extracted values |
|  |  |  | 3 | RVSP: 21 (1); 35 (1); 47 (1) | No extracted values |
|  |  |  | 1 | Tricuspid Regurgitation: tr | No extracted values |
|  |  |  | 4 | WMA: wall motion (1); septal bounce (1); preserved (1); dyskinesis of the anteroseptum (1) | No extracted values |
|  |  | Model failed to correct label values described in report | 20 | Model extracted completely different values with probability score < 0.1 | |
|  |  |  | 34 | Model extracted same annotated values but with probability score < 0.1 | |
| Cardiac catheterization | BioclinicalBERT | Model and manual identified different values | 4 | Diagonal 1: 99%; 30%; 30%; 50% | Diagonal 1: 50%; 20%; 30%; 99% |
|  |  |  | 1 | Diagonal 2: 95% | Diagonal 2: 90% |
|  |  |  | 12 | LAD: luminal irregularities; 60%; luminal irregularities; patent; 40%; 60%; minor plaquing; 50%; 60%; 70%; luminal irregularities; no significant disease | LAD: 50%; 90%; 20%; mild disease; moderate diffuse plaque; 90%; without high grade stenosis; 99%; 70%; 80%; patent; 99% |
|  |  |  | 9 | LCX: 99%; 70%; patent; 40%; 50%; 99%;luminal irregularities; patent; patent | LCX: 50%; 80%; 50%; 80%; 90%; 90%; 80%; 50%; 80% |
|  |  |  | 2 | Left main: some ostial narrowing; 40% | Left main: 70%; 50% |
|  |  |  | 3 | OM1: 90%; 80%; 99% | OM1: 99%; 90%; occluded |
|  |  |  | 2 | OM2: 80%; 99% | OM2: patent; 60% |
|  |  |  | 1 | PLV: 5 | PLV: diffusely diseased |
|  |  |  | 7 | RCA: No significant obstructive disease; 60%; 30%; 70%; 80%; 95%; 99% | RCA: luminal irregularities; 90%; 50%; 40%; 60%; 90%; occlusion |
|  |  | Model extracted values not described in report | 2 | Diagonal 1: 30%; mild diffuse disease | No extracted values |
|  |  |  | 2 | Diagonal 2: ruptured plaque; mild diffuse disease | No extracted values |
|  |  |  | 1 | Diagonal 3: 80% | No extracted values |
|  |  |  | 5 | LCX: No significant stenoses; 90%; 70%; 80%; 90% | No extracted values |
|  |  |  | 9 | OM2: patent (4); diffuse disease; luminal irregularities (2); 80%; no significant obstructive disease | No extracted values |
|  |  |  | 3 | PDA: 80%; spontaneous dissection; without any focal stenotic sites | No extracted values |
|  |  | Model failed to correct label values described in report | 14 | Model extracted completely different values with probability score < 0.1 | |
|  |  |  | 27 | Model extracted same annotated values but with probability score < 0.1 | |
|  | BART-Large-CNN | Model and manual identified different values | 5 | Diagonal 1: 30%; 50%; 60%; 95%; 99% | Diagonal 1: patent; 20%; 99%; 80%; 100%; 50% |
|  |  |  | 1 | Diagonal 2: 95% | Diagonal 2: 90% |
|  |  |  | 9 | LAD: 50%; luminal irregularities; luminal irregularities; 100%; 50%; 60%; 60%; no significant disease; no significant disease | LAD: moderate diffuse plaque; mild disease; patent; 70%; 99%; 90%; 99%; 99%; mild ostial LAD disease |
|  |  |  | 6 | LCX: chronically occluded; patent; patent; luminal irregularities; 40%; 50% | LCX: 50%; 80%; 50%; 80%; 80%; 90% |
|  |  |  | 1 | Left Main: no significant coronary artery disease | Left Main: patent |
|  |  |  |  | OM1: 80% | OM1: subtotally occluded |
|  |  |  | 2 | PLV: mild; subtotally occluded | PLV: luminal irregularities; luminal irregularities |
|  |  |  | 7 | RCA: luminal irregularities; 45%; 60%; 70%; 95%; 99%; no significant obstructive disease | RCA: no significant obstructive disease; luminal irregularities; 90%; 40%; 90%; occlusion; luminal irrregularities |
|  |  | Model extracted values not described in report | 1 | Diagonal 1: 80% | No extracted values |
|  |  |  | 2 | Diagonal 2: mild diffuse disease; mild ostial disease | No extracted values |
|  |  |  | 5 | LCX: free of significant disease; Anomalous; 70%; 80%; 90% | No extracted values |
|  |  |  | 2 | OM2: luminal irregularities; 80% | No extracted values |
|  |  |  | 1 | OM4: patent | No extracted values |
|  |  |  | 1 | OM5: patent | No extracted values |
|  |  |  | 1 | PDA: 80% | No extracted values |
|  |  |  | 1 | PLV: luminal irregularities | No extracted values |
|  |  |  | 1 | RCA: normal | No extracted values |
|  |  | Model failed to correct label values described in report | 11 | Model extracted completely different values with probability score < 0.1 | |
|  |  |  | 26 | Model extracted same annotated values but with probability score < 0.1 | |

Table S10. Examples of echocardiography reports and corresponding manual annotated results and output results generated by the BioclinicalBERT and BART-Large-CNN models with zero-shot learning and fine-tuning at epoch of 8.

| De-identified report (1490, 1497) | Extracted category | Manual annotated results | BioclinicalBERT with fine-tuning | | BioclinicalBERT with zero-shot learning | | BART-Large-CNN with fine-tuning | | BART-Large-CNN with zero-shot learning | |
| --- | --- | --- | --- | --- | --- | --- | --- | --- | --- | --- |
|  |  |  | Extracted value | Score | Extracted value | Score | Extracted value | Score | Extracted value | Score |
| The left ventricle is moderately dilated. Left ventricular ejection fraction is severely reduced (<20%) with global hypokinesis. Moderate diastolic dysfunction. The right ventricle exhibits moderate dilation with preserved function. Moderate atrial enlargement. Moderately severe mitral regurgitation (3+) is present. The aortic valve is not well visualized. Mild aortic regurgitation (1+) is present. No significant AS. Moderate TR. Mild pulmonary hypertension. IVC is dilated w/o respiratory variation. | Aortic regurgitation | mild | mild | 1.00 | hypokinesis | 6.6e-4 | mild | 1.00 | dilation with preserved function. Moderate atrial | 2.9e-4 |
|  | Aortic stenosis | no | no | 1.00 | hypokinesis | 6.6e-4 | no | 1.00 | dilation with preserved function. Moderate atrial | 2.9e-4 |
|  | Diastolic dysfunction | moderate | moderate | 1.00 | hypokinesis | 5.7e-4 | moderate | 1.00 | dilation with preserved function. Moderate atrial | 2.9e-4 |
|  | LAE | moderate | moderate | 1.00 | hypokinesis | 5.5e-4 | moderate | 0.99 |  |  |
|  | LV size | moderately dilated | moderately dilated | 1.00 | aortic | 4.1e-4 | moderately dilated | 1.00 | dilation with preserved function. Moderate atrial | 2.9e-4 |
|  | LVEF | <20% | <20% | 1.00 | aortic | 4.1e-4 | <20% | 1.0 | dilation with preserved function. moderate atrial | 3.0e-4 |
|  | LVH | null | no significant as. moderate tr. mild pulmonary hypertension | 1.5e-21 | aortic | 4.2e-4 | global hypokinesis | 2.2e-16 | dilation with preserved function. moderate atrial | 3.0e-4 |
|  | Mitral regurgitation | moderately severe | moderately severe | 1.00 | hypokinesis | 6.7e-4 | moderately severe | 1.00 | dilation with preserved function. Moderate atrial | 2.8e-4 |
|  | Mitral stenosis | null | moderately severe | 6.0e-22 | hypokinesis | 5.3e-4 | moderate dilation with preserved function. moderate atrial enlargement | 3.0e-17 | dilation with preserved function. Moderate atrial | 2.9e-4 |
|  | Pericardial effusion | null | hypertension. ivc is dilated | 1.6e-21 | hypokinesis | 6.5e-4 | dilated | 5.0e-16 | dilation with preserved function. Moderate atrial | 2.9e-4 |
|  | Prosthetic aortic valve | null | aortic valve is not well visualized. mild aortic | 7.3e-22 | hypokinesis | 6.4-e4 | not well visualized. mild aortic regurgitation | 1.3e-16 | dilation with preserved function. Moderate atrial | 2.8e-4 |
|  | Prosthetic mitral valve | null | 20%) with global hypokinesis | 5.9e-22 | hypokinesis | 5.2e-4 | global hypokinesis. moderate diastolic dysfunction | 3.2e-17 | dilation with preserved function. Moderate atrial | 2.7e-4 |
|  | Prosthetic tricuspid valve | null | regurgitation (1+) is present. no significant as. moderate | 4.4e-22 | hypokinesis | 5.4e-4 | moderate tr | 1.9e-17 | dilation with preserved function. Moderate atrial | 2.9e-4 |
|  | Prosthetic pulmonic valve | null | 20%) with global hypokinesis | 4.8e-22 | hypokinesis | 5.2e-4 | is dilated w/o respiratory variation | 1.3e-17 | dilation with preserved function. Moderate atrial | 2.8e-4 |
|  | Pulmonic HTN | mild | mild | 1.00 | aortic | 4.1e-4 | mild | 1.00 | dilation with preserved function. Moderate atrial | 2.9e-4 |
|  | Pulmonic regurgitation | null | no significant as. moderate | 4.2e-22 | hypokinesis | 5.4e-4 | mild | 5.5e-17 | dilation with preserved function. Moderate atrial | 3.0e-4 |
|  | Pulmonic stenosis | null | mild pulmonary hypertension. ivc is dilated | 4.8e-22 | hypokinesis | 5.3e-4 | is dilated | 1.9e-17 | dilation with preserved function. Moderate atrial | 3.0e-4 |
|  | RA pressure | null | dilated | 3.1e-22 | aortic | 3.0e-4 |  |  | dilation with preserved function. Moderate atrial | 2.9e-4 |
|  | RAE | moderate | moderate | 1.00 | hypokinesis | 5.5e-4 | moderate | 2.2e-7 | dilation with preserved function. Moderate atrial | 3.0e-4 |
|  | RV function | preserved | preserved | 1.00 | hypokinesis | 5.3e-4 | preserved | 1.00 | dilation with preserved function. Moderate atrial | 2.9e-4 |
|  | RV size | moderate dilation | moderate dilation | 1.00 | aortic | 4.2e-4 | moderate dilation | 1.00 | dilation with preserved function. Moderate atrial | 2.9e-4 |
|  | RVSP | null | w/o respiratory variation | 4.0e-22 | aortic | 4.2e-4 | moderate | 1.3e-12 | dilation with preserved function. Moderate atrial | 2.9e-4 |
|  | Tricuspid regurgitation | moderate | moderate | 1.00 | hypokinesis | 5.4e-4 | moderate | 1.00 | dilation with preserved function. Moderate atrial | 2.9e-4 |
|  | Tricuspid stenosis | null | regurgitation (1+) is present. no significant as. moderate | 1.1e-21 | hypokinesis | 5.3e-4 | no significant | 1.7e-17 | dilation with preserved function. Moderate atrial | 3.0e-4 |
|  | WMA | global hypokinesis | global hypokinesis | 1.00 | aortic | 2.8e-4 | global hypokinesis | 1.00 | dilation with preserved function. Moderate atrial | 3.0e-4 |
| NSR at 65/min Normal left ventricular wall thickness. Normal left ventricular size and normal systolic function with an estimated ejection fraction of 60-65 %. Normal diastolic function. Mild biatrial enlargement Structurally normal mitral valve without stenosis or regurgitation. Structurally normal trileaflet aortic valve. Trace to mild aortic regurgitation. No aortic stenosis. Mild tricuspid regurgitation with an estimated PA systolic pressure of 17 mmHg plus estimated RA pressure. No evidence of significant pericardial effusion. | Aortic regurgitation | trace to mild | trace to mild | 1.00 | aortic stenosis. Mild tricuspid | 3.4e-4 | trace to mild | 1.00 | pericardial effusion | 2.1e-4 |
|  | Aortic stenosis | no | no | 1.00 | aortic stenosis. Mild tricuspid | 3.4e-4 | no | 1.00 | 65 %. Normal diastolic function. Mild | 1.2e-4 |
|  | Diastolic dysfunction | normal | normal | 1.00 | aortic stenosis. Mild tricuspid | 3.2e-4 | normal | 1.00 | trileaflet aortic | 1.2e-4 |
|  | LAE | mild | mild | 1.00 | aortic stenosis. Mild tricuspid | 3.4e-4 | mild | 1.00 | mild | 2.1e-4 |
|  | LV size | normal | normal | 1.00 | aortic stenosis. Mild tricuspid | 3.2e-4 | normal | 1.00 | pericardial effusion | 2.0e-4 |
|  | LVEF | 60-65% | 60-65% | 1.00 | aortic stenosis. Mild tricuspid | 3.2e-4 | 60-65% | 1.00 | trileaflet aortic | 1.2e-4 |
|  | LVH | normal | normal | 1.00 | aortic stenosis. Mild tricuspid | 3.3e-4 | normal | 1.00 | Normal diastolic function. Mild | 1.2e-4 |
|  | Mitral regurgitation | without | without | 1.00 | aortic stenosis. Mild tricuspid | 3.4e-4 | without | 1.00 | pericardial effusion | 2.0e-4 |
|  | Mitral stenosis | without | without | 1.00 | aortic stenosis. Mild tricuspid | 3.4e-4 | without | 1.00 | pericardial effusion | 2.0e-4 |
|  | Pericardial effusion | no | no | 1.00 | aortic stenosis. Mild tricuspid | 3.2e-4 | no | 1.00 | trileaflet aortic | 1.2e-4 |
|  | Prosthetic aortic valve | null | aortic valve. Trace to mild aortic | 7.7e-22 | aortic stenosis. Mild tricuspid | 3.4-e4 | Structurally normal trileaflet aortic valve | 5.4e-17 | pericardial effusion | 2.0e-4 |
|  | Prosthetic mitral valve | null | 65 %. Normal diastolic function. Mild biatrial | 6.4e-22 | aortic stenosis. Mild tricuspid | 2.4e-4 | without | 7.7e-17 | pericardial effusion | 2.1e-4 |
|  | Prosthetic tricuspid valve | null | 65 %. Normal diastolic function. Mild biatrial | 4.2e-22 | aortic stenosis. Mild tricuspid | 2.3e-4 | No evidence of significant pericardial effusion | 2.6e-17 | pericardial effusion | 2.0e-4 |
|  | Prosthetic pulmonic valve | null | of 17 mmHg | 5.3e-22 | aortic stenosis. Mild tricuspid | 3.2e-4 | No evidence of significant pericardial effusion | 3.2e-17 | pericardial effusion | 2.e-4 |
|  | Pulmonic HTN | null | enlargement | 1.3e-16 | aortic stenosis. Mild tricuspid | 3.3e-4 | enlargement | 1.3e-16 |  |  |
|  | Pulmonic regurgitation | null | regurgitation with an estimated PA systolic pressure of 17 | 1.4e-21 | aortic stenosis. Mild tricuspid | 3.2e-4 | without | 5.1e-15 | pericardial effusion | 2.1e-4 |
|  | Pulmonic stenosis | null | regurgitation with an estimated PA systolic pressure of 17 | 1.3e-21 | aortic stenosis. Mild tricuspid | 3.2e-4 | No evidence of significant pericardial effusion | 1.9e-17 | 65 %. Normal diastolic function. Mild | 1.2e-4 |
|  | RA pressure | null | plus | 2.1e-21 | aortic stenosis. Mild tricuspid | 3.2e-4 | 17 | 1.4e-14 | pericardial effusion | 2.0e-4 |
|  | RAE | mild | mild | 1.00 | aortic stenosis. Mild tricuspid | 3.4e-4 | mild | 1.00 | mild | 2.1e-4 |
|  | RV function | null | Normal diastolic function. Mild | 7.4e-22 | aortic stenosis. Mild tricuspid | 3.3e-4 | of 17 | 3.6e-17 | trileaflet aortic | 1.2e-4 |
|  | RV size | null | biatrial enlargement | 3.3e-16 | aortic stenosis. Mild tricuspid | 3.2e-4 | biatrial enlargement | 3.3e-16 | pericardial effusion | 2.0e-4 |
|  | RVSP | 17 | 17 | 1.00 | aortic stenosis. Mild tricuspid | 3.2e-4 | 17 | 1.00 | pericardial effusion | 2.1e-4 |
|  | Tricuspid regurgitation | mild | mild | 1.00 | aortic | 3.2e-4 | mild | 1.00 | Structurally normal trileaflet aortic | 2.0e-4 |
|  | Tricuspid stenosis | null | No aortic stenosis. Mild tricuspid | 1.0e-21 | Aortic | 2.2e-4 | without | 3.0e-17 | pericardial effusion | 2.0e-4 |
|  | WMA | null | 65 %. Normal diastolic function. Mild biatrial | 1.4e-21 | aortic stenosis. Mild tricuspid | 3.2e-4 | normal | 1.6e-16 | 65 %. Normal diastolic function. Mild | 1.2e-4 |

Table S11. Examples of cardiac catheterization reports and corresponding manual annotated results and output results generated by the BioclinicalBERT and BART-Large-CNN models with zero-shot learning and fine-tuning at epoch of 8.

| De-identified report (3296,4696) | Extracted category | Manual annotated results | BioclinicalBERT with fine-tuning | | BioclinicalBERT with zero-shot learning | | BART-Large-CNN with fine-tuning | | BART-Large-CNN with zero-shot learning | |
| --- | --- | --- | --- | --- | --- | --- | --- | --- | --- | --- |
|  |  |  | Extracted value | Score | Extracted value | Score | Extracted value | Score | Extracted value | Score |
| Left Main Artery: mild luminal irregularities Left Anterior Descending Artery: medium caliber, mildly calcified, proximal and mid 20%, distal myocardial bridging Diagonal-1 medium caliber, long vessel, ostial 20% Ramus Intermedius Artery: small caliber, ostial 20% Circumflex Artery: medium caliber, proximal 20% Marginal-1 miniscule, patent Marginal-2 medium caliber, proximal 30% Right Coronary Artery: medium to large caliber, proximal 20%, mid 30-40% PDA small to medium caliber, ostial 30% RPLB-1, 2, 3 very small caliber, patent vessels RPLB-4 small to medium caliber, ostial 30% | Left Main | luminal irregularities | luminal irregularities | 1.00 | myocardial bridging | 2.4-e4 | luminal irregularities | 1.00 | coronary artery: | 1.1e-4 |
|  | LAD | 20% | 20% | 1.00 | myocardial bridging | 2.3e-4 | 20% | 1.00 | coronary artery: | 1.1e-4 |
|  | Diagonal 1 | 20% | 20% | 1.00 | myocardial bridging | 2.3e-4 | 20% | 1.00 | coronary artery: | 1.1e-4 |
|  | Diagonal 2 | null | myocardial bridging | 2.0e-20 | myocardial bridging | 2.3e-4 | 20% | 2.4e-16 | coronary artery: | 1.1e-4 |
|  | Diagonal 3 | null | myocardial bridging | 6.9e-21 | myocardial bridging | 2.3e-4 | 20% | 1.7e-17 | coronary artery: | 1.1e-4 |
|  | Ramus | 20% | 20% | 1.00 | myocardial bridging | 2.3e-4 | 20% | 1.00 | coronary artery: | 1.1e-4 |
|  | LCX | 20% | 20% | 1.00 | myocardial bridging | 2.2e-4 | 20% | 1.00 | coronary artery: | 1.1e-4 |
|  | OM1 | patent | patent | 1.00 | myocardial bridging | 9.1e-5 | patent | 1.00 | coronary artery: | 1.1e-4 |
|  | OM2 | 30% | 30% | 1.00 | myocardial bridging | 9.1e-5 | 30% | 1.00 | coronary artery: | 1.1e-4 |
|  | OM3 | null | rplb-1 2 3 very small caliber patent | 2.5e-20 | myocardial bridging | 9.1e-5 | 30% | 3.3e-17 | coronary artery: | 1.1e-4 |
|  | OM4 | null | rplb-1 2 3 very small caliber patent | 2.0e-20 | myocardial bridging | 1.4e-4 | 30% | 8.3e-18 | coronary artery: | 1.1e-4 |
|  | OM5 | null | rplb-1 2 3 very small caliber patent | 9.8e-21 | myocardial bridging | 9.1e-5 | 30% | 7.7e-18 | coronary artery: | 1.1e-4 |
|  | RCA | 40% | 40% | 1.00 | myocardial bridging | 2.3e-4 | 40% | 1.00 | coronary artery: | 1.1e-4 |
|  | PDA | 30% | 30% | 1.00 | myocardial bridging | 2.3e-4 | 30% | 1.00 | coronary artery: | 1.1e-4 |
|  | PLV | 30% | 30% | 1.00 | myocardial bridging | 2.2e-4 | 30% | 1.00 | coronary artery: | 1.1e-4 |
|  | LIMA-LAD | null | 30% | 4.9e-21 | myocardial bridging | 2.2e-4 | myocardial | 6.5e-19 | coronary artery: | 2.0e-4 |
|  | LIMA-Diag | null | 30% | 3.8e-21 | myocardial bridging | 2.2e-4 | proximal and mid 20% distal myocardial bridging | 2.4e-19 | coronary artery: | 2.0e-4 |
|  | LIMA-OM | null | 30% | 5.2e-21 | myocardial bridging | 2.2e-4 | myocardial bridging | 7.0e-20 | coronary artery: | 1.1e-4 |
|  | LIMA-Ramus | null | 30% | 3.8e-21 | myocardial bridging | 2.2e-4 | myocardial | 4.2e-20 | coronary artery: | 2.0e-4 |
|  | SVGs | null | 30% | 5.6e-21 | myocardial bridging | 2.2e-4 | myocardial bridging | 4.7e-19 | coronary artery: | 1.1e-4 |
|  | SVG-OM1 | null | miniscule paten | 1.1e-20 | myocardial bridging | 1.4e-4 | myocardial | 6.8e-20 | coronary artery: | 1.1e-4 |
|  | SVG-OM2 | null | miniscule patent marginal-2 medium caliber proximal 30% | 1.4e-20 | myocardial bridging | 2.2e-4 | myocardial bridging | 2.0e-19 | coronary artery: | 1.1e-4 |
|  | SVG-OM3 | null | 30% | 6.7e-21 | myocardial bridging | 1.4e-4 | 30% right coronary | 1.4e-19 | coronary artery: | 1.1e-4 |
|  | SVG-OM4 | null | 30% | 7.5e-21 | myocardial bridging | 2.2e-4 | 30% right coronary | 9.5e-20 | coronary artery: | 1.1e-4 |
|  | SVG-LCX | null | circumflex artery: medium caliber proximal 20% | 7.6e-21 | myocardial bridging | 2.2e-4 | distal myocardial bridging | 3.6e-20 | coronary artery: | 1.1e-4 |
|  | SVG-D1 | null | 30% | 7.2e-21 | myocardial bridging | 2.2e-4 | proximal and mid 20% distal myocardial bridging | 3.6e-20 | coronary artery: | 2.0e-4 |
|  | SVG-D2 | null | 30% | 9.9e-21 | myocardial bridging | 2.2e-4 | distal myocardial bridging | 5.2e-20 | coronary artery: | 2.0e-4 |
|  | SVG-Ramus | null | intermedius artery: small caliber ostial 20% | 1.2e-20 | myocardial bridging | 2.3e-4 | proximal and mid 20% distal myocardial bridging | 2.0e-20 | coronary artery: | 1.1e-4 |
|  | SVG-RCA | null | 40% | 3.6e-21 | myocardial bridging | 2.3e-4 | proximal and mid 20% distal myocardial bridging | 7.3e-20 | coronary artery: | 1.1e-4 |
|  | SVG-PDA | null | ostial 30% | 1.0e-20 | myocardial bridging | 2.3e-4 | proximal and mid 20% distal myocardial bridging | 1.1e-19 | coronary artery: | 1.1e-4 |
|  | SVG-PLV | null | rplb-1 2 3 very small caliber patent | 8.5e-21 | myocardial bridging | 2.2e-4 | myocardial bridging | 2.9e-19 | coronary artery: | 1.1e-4 |
|  | SVG-LAD | null | 20% | 2.8e-21 | myocardial bridging | 2.3e-4 | proximal and mid 20% distal myocardial bridging | 4.5e-20 | coronary artery: | 1.1e-4 |
|  | SVG-AM | null | 30% | 4.9e-21 | myocardial bridging | 2.2e-4 | myocardial bridging | 3.0e-20 | coronary artery: | 1.1e-4 |
|  | SVG-LM | null | 30% | 6.0e-21 | myocardial bridging | 2.3e-4 | distal myocardial bridging | 2.3e-20 | coronary artery: | 1.1e-4 |
|  | RIMA-LAD | null | 30% | 7.9e-21 | myocardial bridging | 2.2e-4 | 20% distal myocardial bridging | 6.9e-20 | coronary artery: | 1.1e-4 |
|  | RIMA-Diag | null | 30% | 6.9e-21 | myocardial bridging | 2.2e-4 | bridging | 2.6e-19 | coronary artery: | 2.0e-4 |
|  | RIMA-OM | null | 30% | 1.0e-20 | myocardial bridging | 2.1e-4 | luminal irregularities left anterior | 1.4e-19 | coronary artery: | 2.0e-4 |
|  | RIMA-Ramus | null | intermedius artery: small caliber ostial 20% | 1.6e-20 | myocardial bridging | 2.3e-4 | 20% distal myocardial bridging | 1.1e-19 | coronary artery: | 2.0e-4 |
|  | RIMA-PLV | null | rplb-1 2 3 very small caliber patent | 2.4e-20 | myocardial bridging | 2.3e-4 | 20% distal myocardial bridging | 1.6e-19 | coronary artery: | 1.1e-4 |
|  | RIMA | null | 30% | 5.4e-21 | myocardial bridging | 2.3e-4 | myocardial bridging | 9.3e-20 | coronary artery: | 1.1e-4 |
|  | Radial-OM | null | 30% | 6.3e-21 | myocardial bridging | 1.4e-4 | luminal irregularities left anterior | 7.2e-21 | coronary artery: | 1.1e-4 |
|  | Radial-LAD | null | 20% | 7.3e-21 | myocardial bridging | 2.2e-4 | luminal irregularities left anterior | 7.1e-21 | coronary artery: | 1.1e-4 |
|  | Radial-Diag | null | 20% | 6.3e-21 | myocardial bridging | 2.1e-4 | luminal irregularities left anterior | 1.6e-20 | coronary artery: | 2.0e-4 |
|  | Radial-Ramus | null | 30% | 4.6e-21 | myocardial bridging | 2.2e-4 | luminal irregularities left anterior | 8.4e-21 | coronary artery: | 2.0e-4 |
|  | Radial-PDA | null | ostial 30% | 5.1e-21 | myocardial bridging | 2.2e-4 | luminal irregularities left anterior | 1.9e-20 | coronary artery: | 1.1e-4 |
|  | Radial-PLV | null | 30% | 4.7e-21 | myocardial bridging | 2.2e-4 | myocardial bridging | 6.1e-21 | coronary artery: | 1.1e-4 |
|  | Radial | null | 30% | 5.5e-21 | myocardial bridging | 2.4e-4 | myocardial bridging | 2.8e-20 | coronary artery: | 1.1e-4 |
| LEFT MAIN: 20% distal stenosis. LEFT ANTERIOR DESCENDING: 30% in-stent restenosis of the mid LAD stent. D1 is small and appears to be "jailed" by the stent. D2 is small with 90% ostial stenosis. D3 is small and appears patent. Distal LAD with 30% stenosis. LEFT CIRCUMFLEX: 20% proximal, 30-40% mid-distal stenoses. LCx stent with 20% in-stent restenosis. Very distal LCx after OM3 with 80% stenosis. OM1 small with 90% ostial stenosis. OM2 small, ~2 mm vessel at best, with 80% proximal stenosis. OM3 with 40% ostial stenosis. Ramus branch is occluded with late filling via left-to-left collaterals. RIGHT CORONARY ARTERY: 50% mid and 20% distal stenoses. Right PDA diffusely diseased, small vessel with multiple sequential 80% stenoses. collaterals. restenosis. | Left Main | 20% | 20% | 1.00 | occluded with late filling via left-to-left collaterals | 9.7e-5 | 20% | 1.00 | circumflex: | 9.1-e5 |
|  | LAD | 30% | 30% | 1.00 | occluded with late filling via left-to-left collaterals | 9.8e-5 | 30% | 1.00 | circumflex: | 1.3e-4 |
|  | Diagonal 1 | null | jailed | 4.4e-17 | occluded with late filling via left-to-left collaterals | 9.6e-5 | jailed | 3.6e-18 | circumflex: | 9.2e-5 |
|  | Diagonal 2 | 90% | 90% | 1.00 | occluded with late filling via left-to-left collaterals | 9.6e-5 | 90% | 1.00 | circumflex: | 9.2e-5 |
|  | Diagonal 3 | patent | patent | 1.00 | occluded with late filling via left-to-left collaterals | 9.6e-5 | patent | 1.00 | circumflex: | 9.2e-5 |
|  | Ramus | occluded | occluded | 1.00 | occluded with late filling via left-to-left collaterals | 9.9e-5 | occluded | 1.00 | circumflex: | 9.2e-5 |
|  | LCX | 80% | 40% | 1.00 | occluded with late filling via left-to-left collaterals | 1.3e-4 | 40% | 1.00 | circumflex: | 9.1e-5 |
|  | OM1 | 90% | 90% | 1.00 | occluded with late filling via left-to-left collaterals | 1.0e-4 | 90% | 1.00 | circumflex: | 1.3e-4 |
|  | OM2 | 80% | 80% | 1.00 | occluded with late filling via left-to-left collaterals | 1.0e-4 | 80% | 1.00 | circumflex: | 1.3e-4 |
|  | OM3 | 40% | 40% | 1.00 | occluded with late filling via left-to-left collaterals | 1.0e-4 | 40% | 1.00 | circumflex: | 1.3e-4 |
|  | OM4 | null | lcx after om3 with 80% | 1.8e-20 | occluded with late filling via left-to-left collaterals | 1.0e-4 | 80% | 1.1e-17 | circumflex: | 9.5e-5 |
|  | OM5 | null | 80% | 1.4e-20 | occluded with late filling via left-to-left collaterals | 1.0e-4 | 80% | 1.7e-18 | circumflex: | 1.3e-4 |
|  | RCA | 50% | 50% | 1.00 | occluded with late filling via left-to-left collaterals | 9.7e-5 | 50% | 1.00 | circumflex: | 9.1e-5 |
|  | PDA | 80% | 80% | 1.00 | occluded with late filling via left-to-left collaterals | 9.6e-5 | 80% | 1.00 | circumflex: | 9.1e-5 |
|  | PLV | null | lcx after om3 with 80% | 3.4e-20 | occluded with late filling via left-to-left collaterals | 1.0e-4 | 80% | 1.1e-15 | circumflex: | 9.1e-5 |
|  | LIMA-LAD | null | 80% | 5.7e-21 | occluded with late filling via left-to-left collaterals | 1.0e-4 | restenosis | 2.5e-20 | circumflex: | 1.3e-4 |
|  | LIMA-Diag | null | 80% | 4.6e-21 | occluded with late filling via left-to-left collaterals | 1.0e-4 | collaterals | 3.3e-21 | circumflex: | 1.3e-4 |
|  | LIMA-OM | null | lcx after om3 with 80% | 6.1e-21 | occluded with late filling via left-to-left collaterals | 1.0e-4 | om2 small ~2 mm vessel at best with 80% | 1.4e-21 | circumflex: | 1.3e-4 |
|  | LIMA-Ramus | null | 80% | 4.4e-21 | occluded with late filling via left-to-left collaterals | 1.3e-4 | collaterals | 2.0e-21 | circumflex: | 1.3e-4 |
|  | SVGs | null | 80% | 1.0e-20 | occluded with late filling via left-to-left collaterals | 1.0e-4 | 80% stenoses. collaterals | 3.0e-19 | circumflex: | 9.1e-5 |
|  | SVG-OM1 | null | om1 small with 90% | 1.4e-20 | occluded with late filling via left-to-left collaterals | 6.8e-5 | 80% stenoses. collaterals | 1.1e-20 | circumflex: | 1.3e-4 |
|  | SVG-OM2 | null | 80% proximal stenosis | 1.9e-20 | occluded with late filling via left-to-left collaterals | 6.8e-5 | 80% stenoses. collaterals | 3.2e-20 | circumflex: | 1.3e-4 |
|  | SVG-OM3 | null | 40% ostial stenosis | 2.6e-20 | occluded with late filling via left-to-left collaterals | 9.7e-5 | 80% stenoses. collaterals | 2.9e-20 | circumflex: | 1.3e-4 |
|  | SVG-OM4 | null | 80% | 1.3e-20 | occluded with late filling via left-to-left collaterals | 9.8e-5 | 80% stenoses. collaterals | 1.8e-20 | circumflex: | 1.3e-4 |
|  | SVG-LCX | null | 80% stenosis | 1.1e-20 | occluded with late filling via left-to-left collaterals | 9.8e-5 | 80% stenoses. collaterals | 7.1e-20 | circumflex: | 1.3e-4 |
|  | SVG-D1 | null | appears to be | 4.5e-20 | occluded with late filling via left-to-left collaterals | 1.0e-4 | 80% stenoses. collaterals | 6.2e-21 | circumflex: | 1.3e-4 |
|  | SVG-D2 | null | 90% ostial stenosis | 1.1e-20 | occluded with late filling via left-to-left collaterals | 1.0e-4 | 80% stenoses. collaterals | 2.0e-20 | circumflex: | 1.3e-4 |
|  | SVG-Ramus | null | occluded | 1.3e-20 | occluded with late filling via left-to-left collaterals | 1.0e-4 | 80% stenoses. collaterals | 3.6e-21 | circumflex: | 1.3e-4 |
|  | SVG-RCA | null | 50% mid and 20% distal stenoses | 7.6e-21 | occluded with late filling via left-to-left collaterals | 1.0e-4 | multiple sequential 80% stenoses. collaterals | 5.2e-21 | circumflex: | 1.3e-4 |
|  | SVG-PDA | null | diffusely diseased small vessel with multiple sequential 80% | 2.6e-20 | occluded with late filling via left-to-left collaterals | 1.0e-4 | 80% stenoses. collaterals | 8.6e-21 | circumflex: | 9.1e-5 |
|  | SVG-PLV | null | 80% | 6.2e-21 | occluded with late filling via left-to-left collaterals | 9.7e-5 | multiple sequential 80% stenoses. collaterals | 5.6e-21 | circumflex: | 1.3e-4 |
|  | SVG-LAD | null | 30% | 7.5e-21 | occluded with late filling via left-to-left collaterals | 9.9e-5 | 80% stenoses. collaterals | 8.5e-21 | circumflex: | 1.3e-4 |
|  | SVG-AM | null | 80% | 5.7e-21 | occluded with late filling via left-to-left collaterals | 9.9e-5 | 80% stenoses. collaterals | 2.4e-21 | circumflex: | 9.0e-5 |
|  | SVG-LM | null | 80% | 6.9e-21 | occluded with late filling via left-to-left collaterals | 1.0e-4 | 80% stenoses. collaterals | 1.7e-21 | circumflex: | 9.0e-5 |
|  | RIMA-LAD | null | 30% | 8.7e-21 | occluded with late filling via left-to-left collaterals | 1.0e-4 | 50% | 7.9e-20 | circumflex: | 1.3e-4 |
|  | RIMA-Diag | null | 80% | 7.7e-21 | occluded with late filling via left-to-left collaterals | 1.0e-4 | 80% stenoses. collaterals | 3.7e-20 | circumflex: | 1.3e-4 |
|  | RIMA-OM | null | 80% | 1.0e-20 | occluded with late filling via left-to-left collaterals | 1.0e-4 | 50% | 2.5e-20 | circumflex: | 1.3e-4 |
|  | RIMA-Ramus | null | occluded | 1.5e-20 | occluded with late filling via left-to-left collaterals | 1.3e-4 | 80% stenoses. collaterals | 3.7e-20 | circumflex: | 1.3e-4 |
|  | RIMA-PLV | null | 80% | 7.0e-21 | occluded with late filling via left-to-left collaterals | 1.0e-4 | 50% mid and 20% distal stenoses. right pda | 7.5e-20 | circumflex: | 1.3e-4 |
|  | RIMA | null | 80% | 8.4e-21 | occluded with late filling via left-to-left collaterals | 9.7e-5 | 50% | 1.6e-19 | circumflex: | 9.0-e5 |
|  | Radial-OM | null | 80% | 7.1e-21 | occluded with late filling via left-to-left collaterals | 1.3e-4 | 80% stenoses. collaterals | 3.6e-22 | circumflex: | 1.3e-4 |
|  | Radial-LAD | null | 80% | 5.1e-21 | occluded with late filling via left-to-left collaterals | 9.8e-5 | left | 6.1e-22 | circumflex: | 1.3e-4 |
|  | Radial-Diag | null | 80% | 4.3e-21 | occluded with late filling via left-to-left collaterals | 1.0e-4 | left | 1.8e-22 | circumflex: | 1.3e-4 |
|  | Radial-Ramus | null | 80% | 4.5e-21 | occluded with late filling via left-to-left collaterals | 1.3e-4 | left | 5.1e-22 | circumflex: | 1.3e-4 |
|  | Radial-PDA | null | diffusely diseased small vessel with multiple sequential 80% | 9.7e-21 | occluded with late filling via left-to-left collaterals | 1.0e-4 | left | 1.8e-21 | circumflex: | 1.3e-4 |
|  | Radial-PLV | null | collaterals | 6.2e-21 | occluded with late filling via left-to-left collaterals | 9.9e-5 | left | 1.3e-21 | circumflex: | 1.3e-4 |
|  | Radial | null | 80% | 6.1e-21 | occluded with late filling via left-to-left collaterals | 9.8e-5 | 80% stenoses. collaterals | 2.0e-21 | circumflex: | 9.1e-5 |
